# ArcA modulates multidrug resistance and compound susceptibility in *Klebsiella pneumoniae* through ArcB-independent regulation of the SMR efflux pump kpnEF

**DOI:** 10.1128/spectrum.01499-25

**Published:** 2025-10-13

**Authors:** Tongtong Fu, Zheng Fan, Yuchen Chen, Zhoufei Li, Hongbo Liu, Bing Du, Xiaohu Cui, Yanling Feng, Hanqing Zhao, Guanhua Xue, Jinghua Cui, Chao Yan, Lin Gan, Junxia Feng, Ziying Xu, Yang Yang, Zihui Yu, Yuehua Ke, Jing Yuan

**Affiliations:** 1 Department of Bacteriology, Capital Center for Children's Health, Capital Medical University, Capital Institute of Pediatrics 36776, Beijing, China; 2 Graduate School of Peking Union Medical College 196536, Beijing, China; The University of Texas at Tyler, Tyler, Texas, USA

**Keywords:** *Klebsiella pneumoniae*, ArcAB, regulator, resistance, SMR efflux pump

## Abstract

*Klebsiella pneumoniae* has become a major clinical and public health threat due to the increasing prevalence of healthcare-associated infections caused by multidrug-resistant strains. In this study, we demonstrated that the deletion of *arcA* of ArcAB two-component system diminished the susceptibility of *K. pneumoniae* to antibiotics, osmotic agents, disinfectants, and structural compounds, which was independent of *arcB*. RNA-seq analysis revealed a marked upregulation of SMR efflux pump genes *kpnEF* in the Δ*arcA* strain compared to the wild-type strain, while the Δ*arcB* strain exhibited no significant changes. Notably, the deletion of *kpnEF* in both Δ*arcA* and wild-type strains abolished their differential susceptibility to antibiotics, osmotic agents, disinfectants, and structural compounds. The EMSA experiments showed that ArcA-P regulated the *kpnEF* transcriptional expression by directly binding to its promoter region. These findings indicated that ArcA could directly modulate the expression of the KpnEF efflux pump independently of its sensor kinase ArcB. Through phosphorylation level detection and gene knockout experiments, we found that ArcA phosphorylation in the Δ*arcB* strain was primarily mediated by the AckA-Pta pathway. This study expanded the function of the ArcAB system and provided a critical theoretical foundation for elucidating the mechanisms underlying bacterial antibiotic resistance in *K. pneumoniae*.

**IMPORTANCE:**

*Klebsiella pneumoniae* is an important opportunistic bacterial pathogen, which can acquire a series of antimicrobial resistance (AMR) genes. The emergence of carbapenem-resistant *K. pneumoniae* (CRKP) posed significant challenges to public health. Polymyxins are often regarded as the last line of defense against CRKP infections. In this study, the deletion of *arcA*, the regulator of the two-component system ArcAB, increased resistance of *K. pneumoniae* to antibiotics and decreased susceptibility to osmotic agents, disinfectants, and structural compounds, which was independent of ArcB. Further experiments have shown that ArcA regulated the expression of the small multidrug resistance (SMR) pump KpnEF through direct binding. This process required ArcA phosphorylation, which was independent of ArcB but dependent on the AckA-Pta pathway. This study deepened the regulatory network of ArcAB and provided a new target for the treatment of *K. pneumoniae*.

## INTRODUCTION

*Klebsiella pneumoniae* is an important opportunistic bacterial pathogen and a leading cause of both nosocomial and community-acquired infections (1). This pathogen exhibits an exceptional ability to acquire a diverse range of antimicrobial resistance (AMR) genes, primarily through the uptake of plasmids and mobile genetic elements (2). This genetic adaptability has led to the emergence of multidrug-resistant (MDR) and extensively drug-resistant (XDR) strains, posing significant challenges to public health. Among these, the rise of carbapenem-resistant *K. pneumoniae* (CRKP) has particularly complicated clinical treatment, as it limits therapeutic options (3). In such cases, polymyxin B and colistin are often employed as the last line of defense against CRKP infections (4). Bacteria, including *K. pneumoniae*, have evolved multiple mechanisms to resist antibiotics. These mechanisms commonly include enzymatic modification and inactivation of antibiotics, modification of antibiotic targets, efflux of antibiotics, and the development of bypass mechanisms that can replace or circumvent drug targets (5).

Antibiotic efflux pumps are key to bacterial resistance and are divided into several families: ATP-binding cassette (ABC), major facilitator superfamily (MFS), resistance/nodulation/cell division (RND), multidrug and toxic compound extrusion (MATE), proteobacterial antimicrobial compound efflux (PACE), and small multidrug resistance (SMR). These pumps use proton motive force (PMF) or ATP to expel antibiotics and toxins from cells (6–9). The SMR family is unique for its small size (100–150 amino acids) and four transmembrane helices (TM1–TM4) (10, 11). The SMR protein family is categorized into three distinct subclasses: small multidrug proteins (SMP), suppressor of groEL mutations, and paired small multidrug resistance (PSMR) proteins. EmrE in *E. coli* and *P. aeruginosa*, SsmE in *S. marcescens*, and AbeS in *A. baumannii* belong to SMP subclass (12–15). The experimentally characterized pairs MdtI/MdtJ, YkkC/YkkD, and EbrA/EbrB are classified within the PSMR subclass (16, 17). The SMR efflux pump KpnEF, a member of the PSMR subclass, functions as a homolog of EbrAB and plays a role in both physiological processes and multidrug resistance within the *K. pneumoniae* (18). SMR pumps are valuable for studying bacterial resistance due to their simple structure and specific function. They also offer potential targets for new antibiotics and resistance-fighting strategies.

The anoxic redox control (or aerobic respiration control) two-component system, “ArcAB system,” consists of regulator ArcA and the sensor kinase ArcB. The “ArcAB system” is involved in the metabolic transition from aerobic respiration to fermentation and anaerobic respiration when bacteria enter the micro-oxygen and anaerobic environment (19). When sensing oxygen consumption, sensor kinase ArcB auto-phosphorylated and trans-phosphorylated regulator ArcA via the ArcBHis292 - ArcBAsp576 - ArcBHis717- ArcAAsp54 process (20–22). ArcA remains capable of phosphorylation through other pathways in the absence of ArcB. In *Shewanella oneidensis* MR-1, ArcS likely functions as the cognate sensor kinase of the Arc system, a role previously attributed to ArcB in *Escherichia coli* (23). The AckA-Pta pathway could modulate the expression of ArcA-P target genes in the absence of ArcB in *E. coli* (24). In *vitro*, the ArcA protein could be phosphorylated by carbamoyl phosphate (25, 26). Phosphorylated ArcA could promote fermentation pathway and inhibit pathways associated with aerobic respiration (27–29). ArcA has been characterized and could regulate a multitude of processes, including response to growth limitation, response to reactive oxygen species and even pathogenesis (19). As a broad regulatory factor, the regulatory functions of ArcA remain to be further explored.

Our previous study found that high-alcohol-producing *K. pneumoniae* (W14), obtained from a patient with auto-brewery syndrome, is a primary causative agent of NAFLD (30). To investigate the mechanism of high ethanol production in W14 and screen for potential therapeutic targets, we constructed knockout strains of the global regulatory factors Fnr, IhfAB, ArcAB, and Crp separately. Subsequently, the ethanol production capacity and antibiotic resistance in these mutant strains were measured. We found that ArcA played a crucial role in the sensitivity of *K. pneumoniae* to antibiotics, osmotic agents, disinfectants, and structural compounds, which was independent of ArcB. The expression of the SMR efflux pump KpnEF was significantly upregulated in the Δ*arcA*, so further deletion of *kpnEF* restored the sensitivity of the Δ*arcA*. Additional experiments demonstrated that ArcA directly binds to the promoter region of *kpnEF*, regulating its expression and thereby influencing the bacterial sensitivity to multiple compounds. These findings enhanced our understanding of the regulatory mechanisms underlying drug resistance and enriched the regulatory network of the ArcAB system in *K. pneumoniae*.

## MATERIALS AND METHODS

### Bacterial strains, plasmids, and primers

The K30 (clinical *Klebsiella pneumoniae*) cKp W14 was utilized as the wild-type strain (WT) in this study. All strains employed for both culture and phenotypic analysis were incubated at a temperature of 37°C in a shaking incubator with a speed of 180–200 rpm. And the culture media used was Luria-Bertani (LB) broth (5 g/L yeast extract, 10 g/L sodium chloride, and 10 g/L tryptone). The addition of 50 µg/mL kanamycin was used for the construction of gene knockout strains and complemented strains. The related strains and plasmids were listed in Table S1. The primers in this study were listed in Table S2.

### Mutant and complementation strains construction

To obtain the Δ*arcA*, Δ*arcB*, Δ*kpnEF,* and Δ*kpnEF*Δ*arcA* mutant strains and related complementation strains, methods by Link et al. and Pan et al. were used (31, 32). Briefly, the flanking regions of *arcA*, *arcB,* and *kpnEF* cluster were ligated into the plasmid pKO3-Km (digested by *Not* I) and the resulting plasmids were transformed into the wild-type strains W14 or the Δ*arcA* strains by electroporation. At 43°C and 37°C, respectively, plasmid was first inserted into the bacterial chromosome and then shed and screened for the correct mutant strain by 5% sucrose plate. For the construction of the *arcA* and *arcB* complementation strain, these genes coding region and their ribosomal binding site were cloned into the plasmid pGEM-T Easy (digested by *Sal* I and *Sph* I). Then these resulting plasmids were transformed into their related mutant strains respectively by electroporation.

### Bacterial growth curves

Growth curves of W14, Δ*arcA*, Δ*arcB*, Δ*arcA*/P*arcA,* and Δ*arcB*/P*arcB* strains were drawn by subculturing in LB broth overnight. Briefly, the overnight cultures of wild type (W14), deletion strains and complementary strains were diluted 1:100 into 20 mL of fresh LB broth and then grown by shaking at 200 rpm at 37°C. OD600 measurements were detected per hour to represent the cell density. Three independent cultures were used for each assay.

### RNA extraction, reverse transcription, RNA-seq, and quantitative real-time PCR

The bacterial suspension was subjected to centrifugation at a speed of 11,340 × *g*, and then bacterial precipitate was resuspended in 100 µL of 1× RNase-free TAE and subsequent extraction of total RNA was performed utilizing RNAprep Pure Cell/Bacteria Kit (Tiangen Biotech, Beijing, China). A quantity of RNA ranging from 0.5 to 1 µg was employed for the process of reverse transcription, wherein cDNA was synthesized utilizing PrimeScript Reverse Transcriptase (TaKaRa, Dalian, China). The qRT-PCR experiment was conducted by employing a 20 µL mixture comprising the SYBR Premix Ex TaqTM II (TaKaRa), cDNA templates, and specific forward and reverse primers. The qRT-PCR reactions were performed on the QuantStudio 5 Real-Time PCR System (Applied Biosystems, USA). The Ct values of the target genes were normalized to the reference gene r*poB*, and the fold-change in gene expression was calculated using the classic 2^(−ΔΔCt) method. RNA sequencing and analysis services were performed by NoVo gene (Beijing, China).

### Minimal inhibit concentration assay

The MICs were determined following the guidelines outlined in the Clinical and Laboratory Standards Institute (CLSI) M07 11th edition and M100 32nd edition. *E. coli* ATCC 25922 was employed as the quality control strain. Overnight cultures were diluted 1:100 into fresh medium and incubated at 37°C with shaking at 220 rpm until the OD600 reached 0.8–1.0. The bacterial strains used for MIC determination showed no significant differences in colony counts. The bacterial suspension was then appropriately diluted and dispensed into 96-well plates. Antibiotics, including cefepime, ceftazidime, kanamycin, ciprofloxacin, streptomycin, erythromycin, rifampicin, colistin E, polymyxin B, and tetracycline, were serially diluted using the twofold broth microdilution method and added to the 96-well plates. For the MICs testing of the above-mentioned antibiotics, cation-adjusted Mueller-Hinton broth (CAMHB) medium was utilized, with each well receiving an inoculum of 5 × 10⁵ CFU. The plates were incubated at 37°C for 20 h, after which the MIC was determined as the lowest antibiotic concentration that completely inhibited visible bacterial growth.

### Structurally related compounds, hospital-based disinfectants, osmotic compounds challenge assays

Various stress assays were conducted as previously described (33). Briefly, mutant, complemented, and WT strains were cultured to mid-exponential phase, and the cultures were then spread onto LB agar plates containing different concentrations of the following compounds: osmotic stress agent NaCl (0.075 M, 0.15 M, 0.25 M, 0.5 M, 0.75 M, 1 M, and 2 M); disinfectants include benzalkonium chloride, chlorhexidine (3.2 µg/mL, 6.4 µg/mL, 12.8 µg/mL, 25.6 µg/mL, 51.2 µg/mL) and triclosan (0.01 µg/mL, 0.05 µg/mL, 0.1 µg/mL, 0.5 µg/mL, 1 µg/mL, 2 µg/mL); structure-related compounds include SDS (1,024 µg/mL, 2,048 µg/mL, 4,096 µg/mL, 8,192 µg/mL, 16,384 µg/mL), ethidium bromide, and acriflavine (2 µg/mL, 8 µg/mL, 64 µg/mL, 128 µg/mL, 256 µg/mL, 512 µg/mL). The results were expressed as the ratio of the number of colony-forming units (CFU) obtained from LB plates containing different concentrations of NaCl, disinfectants, and structure-related compounds to the number of CFU obtained from control plates (LB agar alone). Each experiment was repeated at least three times.

### Protein expression and purification

To construct the ArcA expression plasmid, the *arcA* gene fragment was amplified from the W14 genome and ligated into the *Nco* I and *Hind* III digested pET28a vector. Transformants were selected on kanamycin plates, and the correct pET28A-His6-ArcA expression plasmid was verified by PCR and sequencing. The confirmed pET28a-His6-ArcA plasmid was then transformed into *E. coli* BL21(DE3). The transformants were cultured in LB medium supplemented with 50 µg/mL kanamycin at 37°C until the OD600 reached 0.6, followed by induction with 0.5 mM IPTG at 37°C for 3 hs. The cells were harvested and lysed by sonication in lysis buffer (25 mM HEPES, 5 mM β-mercaptoethanol, 500 mM NaCl, and 10% glycerol, pH 7.8). The His-tagged fusion protein was purified using nickel-nitrilotriacetic acid (Ni-NTA) agarose resin (Qiagen, Beijing, China) and eluted with elution buffer (25 mM HEPES, 5 mM β-mercaptoethanol, 500 mM NaCl, 10% glycerol, and 300 mM imidazole, pH 7.8). The target protein was stored at −80°C until further use. The concentration of the purified protein was determined by SDS-PAGE and quantified using the bicinchoninic acid (BCA) assay with a standard protein as the reference.

### Electrophoretic mobility shift assay

The EMSA was performed following a previously described method with slight modifications (34). We amplified the DNA fragment of the *kpnEF* promoter region using W14 as the template. The *kpnEF* promoter (50 ng) DNA fragment was pre-incubated with increasing concentrations of purified ArcA protein in a binding buffer (20 µL) containing 10 mM Tris–HCl (pH 8.0), 1 mM EDTA, 1 mM DTT, 1.5 mg/mL poly IC, 50 mM KCl, 50 µg/mL BSA, and 10% glycerol, supplemented with potassium lithium acetyl phosphate (20 nM), at ambient temperature for 30 min. An 8% polyacrylamide gel was pre-electrophoresed in 1 × Tris-borate-EDTA buffer (0.044 M Tris, 0.044 M boric acid, and 0.001 M EDTA, pH 8.0) for 1 h to remove impurities. After loading the samples, electrophoresis was conducted on ice for 1 h and 40 min. Following electrophoresis, the gel was stained with 0.5 µg/mL ethidium bromide. Imaging was performed using a gel documentation system (Bio-Rad, Hercules, CA, USA).

### Phos-assay western blotting

The phos assay reagent (Vazyme, Nanjing, China) was added to the 8.5% SDS-PAGE separating gel for phosphorylation level detection. Cells were lysed in SDS-loading buffer (2% β-mercaptoethanol) and boiled for 10 min. Protein concentrations were determined by BCA assay, and 20 µg of protein was separated by Phos-assay SDS-PAGE. After transfer to PVDF membranes, blots were blocked with 5% BSA/TBST and probed with anti-His (1:2,000, Sigma, USA) at 4°C overnight, followed by HRP-conjugated secondary antibody anti-Mousse IgG (1:2,000, Promega, USA). Signals were detected by Immobilon Western HRP.

### Co-immunoprecipitation/MS

The Δ*arcB* mutant strain complemented with His-tagged ArcA was lysed in NP-40 buffer (50 mM Tris-HCl pH 7.4, 150 mM NaCl, 1% NP-40, protease inhibitors). Lysates were split into two aliquots: one incubated with anti-His beads (Sigma, USA), the other with mouse IgG1 isotype control (Thermo, USA) for 4 h at 4°C. Both groups used Protein G magnetic beads and identical wash conditions. After washing five times with NP-40 buffer, bound proteins were eluted with 2 × Laemmli buffer. IgG controls were processed in parallel. Samples were trypsin-digested (Promega, USA) and analyzed by LC-MS/MS (Q Exactive HF-X). Data were searched against the UniProt Escherichia coli database (2023 release) using PEAKS Studio with a 1% FDR threshold. Specific interactors required ≥2 unique peptides and Fold Change (His/IgG) ≥2.

### Statistical analysis

Three biological replicates were performed for each experiment. Statistical analysis was conducted using GraphPad Prism (version 5.0, USA), and the data were presented as mean ± standard deviation (SD). Statistical significance was determined by one-way ANOVA, with *P* < 0.05, *P* < 0.01, and *P* < 0.001, *P* < 0.0001 indicating significant differences. Following one-way analysis of variance (ANOVA) which revealed significant differences, we performed all pairwise comparisons using Tukey’s Honest Significant Difference (HSD) test. Growth curve statistical analyses were performed using SPSS software Statistics 27.0.1 with repeated-measures ANOVA.

## RESULTS

### ArcA influenced the susceptibility of *K. pneumoniae* to multiple antibiotics, and this effect was independent of *arcB*

To investigate whether the two-component system ArcA-ArcB affected the antibiotic resistance of *K. pneumoniae*, the minimum inhibitory concentrations (MICs) of WT, Δ*arcA*, Δ*arcB*, Δ*arcA*/P*arcA*, and Δ*arcB*/P*arcB* strains were determined against cefepime, ceftazidime, kanamycin, streptomycin, ciprofloxacin, colistin E, erythromycin, polymyxin B, rifampicin, and tetracycline. The MICs of the Δ*arcA* strains for the above antibiotics were two-, four-, four-, one-, two-, four-, one-, four-, one-, and twofold higher than those of the wild-type strains, respectively (Table 1). The antibiotic susceptibility of the Δ*arcA* strains was restored in the Δ*arcA*/P*arcA* strains. In contrast, the MICs of the Δ*arcB* strains were consistent with those of the WT strains (Table 1).

**TABLE 1:** The MICs of WT, Δ*arcA***,** Δ*arcA***/**P*arcA*, Δ*arcB,* and Δ*arcB***/**P*arcB* strains to antibiotics

	MIC (µg/mL)				
Antibiotics	WT strain	Δ*arcA*	Δ*arcA*/P*arcA*	Δ*arcB*	Δ*arcB*/P*arcB*
Cefepime	0.125	0.25	0.125	0.125	0.125
Ceftazidime	0.063	0.125	0.063	0.063	0.063
Kanamycin	0.625	2.5	0.625	0.625	0.625
Streptomycin	>62.5	>62.5	>62.5	>62.5	>62.5
Ciprofloxacin	0.001	0.002	0.001	0.001	0.001
Colistin	1	4	1	1	1
Erythromycin	1.25	1.25	1.25	1.25	1.25
Polymyxin B	1	4	1	1	1
Rifampin	0.625	0.625	0.625	0.625	0.625
Tetracycline	3.125	6.25	3.125	3.125	3.125

### ArcA decreased the susceptibility of *K. pneumoniae* to osmotic stress compound, hospital disinfectants, and structurally related compounds by an *arcB***-**independent manner

**Fig 1:**
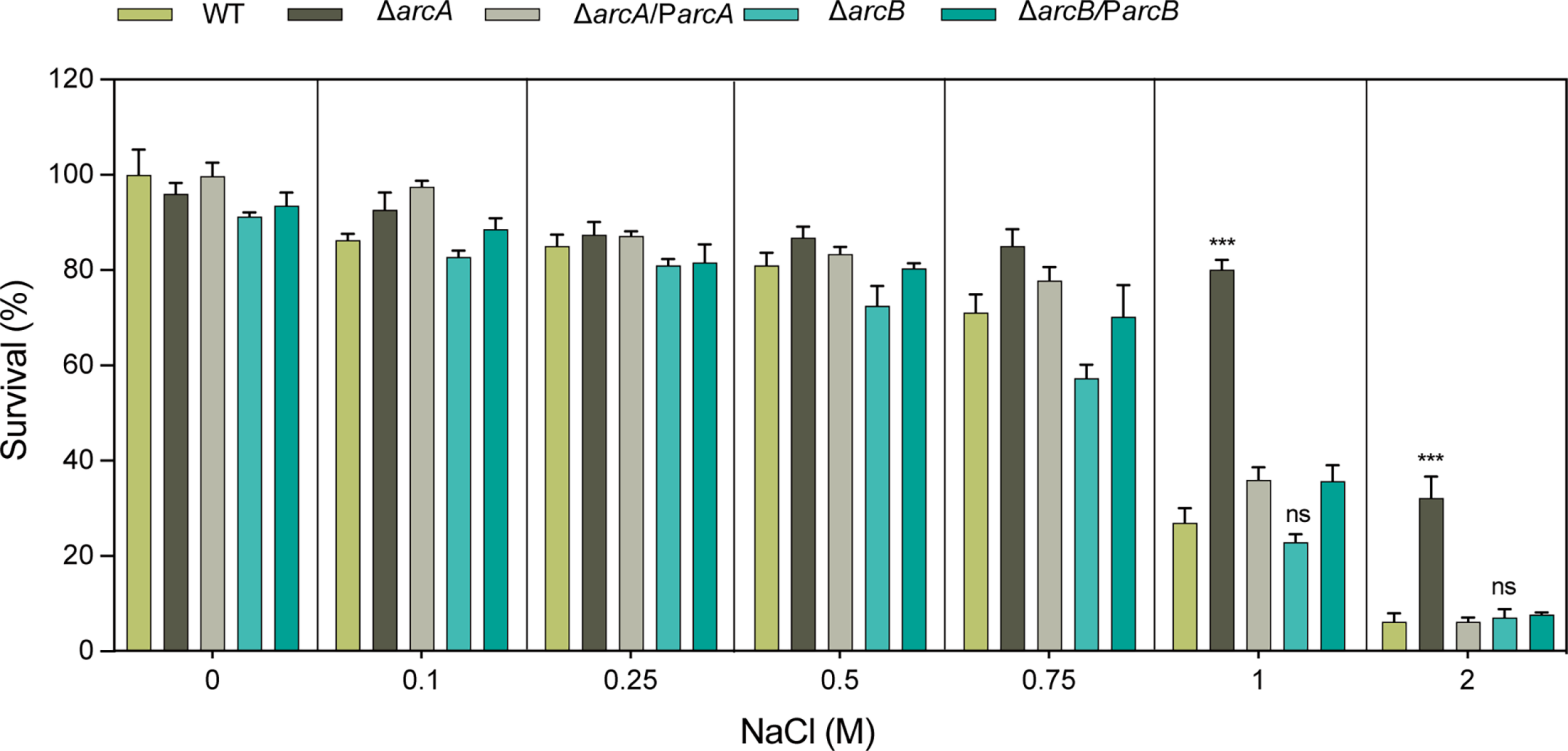
The susceptibility of the WT, Δ*arcA***,** Δ*arcB***,** Δ*arcA***/**P*arcA,* and Δ*arcB***/**P*arcB* strains to osmotic stress agent. Sensitivities of WT, Δ*arcA*, ΔarcB, Δ*arcA*/P*arcA,* and Δ*arcB*/P*arcB* strains to different concentrations (0.075, 0.15, 0.25, 0.5, 0.75, 1.0, and 2.0 M) of NaCl. The survival ability of the Δ*arcA* in 1 M NaCl was 2.65-fold greater, and in 2 M NaCl, it was 4.38-fold greater compared to the WT strain. The resistance percentages to different concentrations of NaCl were calculated by comparing the numbers of surviving cells to those in LB medium alone. Asterisks indicate statistically significant differences compared to the WT strain. Each experiment was performed in triplicate. *P < 0.05, **P < 0.01, ***P < 0.001, ****P < 0.0001, analyzed with one-way ANOVA.

In this study, the susceptibilities of the WT, Δ*arcA*, Δ*arcB*, Δ*arcA*/P*arcA,* and Δ*arcB*/P*arcB* strains to different concentrations of osmotic stress compound, disinfectants, and structurally related compounds were evaluated. Under NaCl conditions (physiological concentration, 150 mM) (33, 35), the viability of the Δ*arcA* was significantly higher than that of the wild type. Specifically, in 1 M NaCl, the growth capacity of the Δ*arcA* was approximately 2.65-fold greater than that of the wild-type, and in 2 M NaCl, it was approximately 4.38-fold greater. This difference was independent of the inoculum size (Fig. 1).

**Fig 2:**
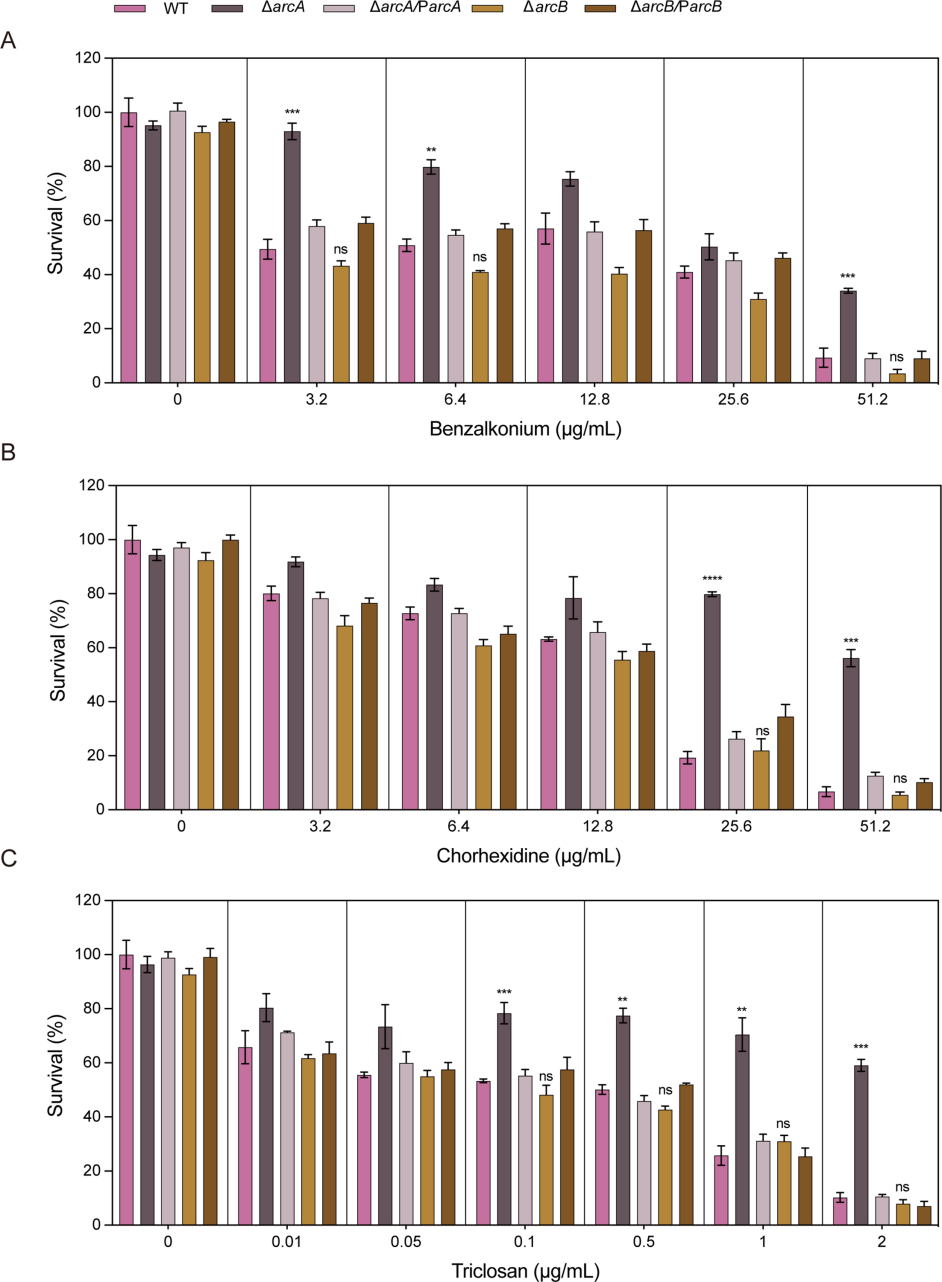
The susceptibility of the WT, Δ*arcA*, Δ*arcB*, Δ*arcA***/**P*arcA,* and Δ*arcB***/**P*arcB* strains to hospital-based disinfectants. (**A**) Sensitivities of the WT, Δ*arcA*, ΔarcB, Δ*arcA*/P*arcA,* and Δ*arcB*/P*arcB* strains to different concentrations (3.2 µg/mL, 6.4 µg/mL, 12.8 µg/mL, 25.6 µg/mL, 51.2 µg/mL) of benzalkonium chloride. The survival ability of the Δ*arcA* in benzalkonium chloride at 51.2 µg/mL was 3.64-fold greater, at 6.4 µg/mL, it was 1.57-fold greater, and at 3.2 µg/mL, it was 1.72-fold greater compared to the WT strain. (**B**) Sensitivities of WT, Δ*arcA*, Δ*arcB*, Δ*arcA*/P*arcA,* and Δ*arcB*/P*arcB* strains to different concentrations (3.2 µg/mL, 6.4 µg/mL, 12.8 µg/mL, 25.6 µg/mL, 51.2 µg/mL) of chlorhexidine. The survival ability of the Δ*arcA* in chlorhexidine at 51.2 µg/mL was 8.35-fold greater, and at 25.6 µg/mL was 3.80-fold greater compared to the WT strain. (**C**) Sensitivities of WT, Δ*arcA*, ΔarcB, Δ*arcA*/P*arcA,* and Δ*arcB*/P*arcB* strains to different concentrations (0.01 µg/mL, 0.05 µg/mL, 0.1 µg/mL, 0.5 µg/mL, 1 µg/mL, 2 µg/mL) of triclosan. The survival ability of the Δ*arcA* in triclosan at 0.1 µg/mL was 1.47-fold, at 0.5 µg/mL was 1.50-fold, at 1 µg/mL was 2.58-fold, and at 2 µg/mL was 5.26-fold greater compared to the WT strain. The resistance percentages to different concentrations of benzalkonium chloride, chlorhexidine, and triclosan were calculated by comparing the numbers of surviving cells to those in LB medium alone. Asterisks indicate statistically significant differences compared to the WT strain. Each experiment was performed in triplicate. *P < 0.05, **P < 0.01, ***P < 0.001, ****P < 0.0001, analyzed with one-way ANOVA.

When cells were exposed to varying concentrations of benzalkonium chloride, it was observed that at 51.2 µg/mL, the total CFU count of the Δ*arcA* was 3.64-fold higher than that of the wild type; at 6.4 µg/mL, the total CFU count of the Δ*arcA* was 1.57-fold higher than that of the wild type, and at 3.2 µg/mL, the total CFU count of the Δ*arcA* was 1.72-fold higher than that of the wild type (Fig. 2A). When cells were exposed to varying concentrations of chlorhexidine, it was observed that at 51.2 µg/mL, the total CFU count of the Δ*arcA* was 8.35-fold higher than that of the wild type, and at 25.6 µg/mL, the total CFU count of the Δ*arcA* was 3.80-fold higher than that of the wild-type strain (Fig. 2B). Similarly, when cells were exposed to 0.1, 0.5, 1, and 2 µg/mL triclosan, the total CFU count of the Δ*arcA* was 1.47-, 1.50-, 2.58-, and 5.26-fold higher than that of the wild type (Fig. 2C).

**Fig 3:**
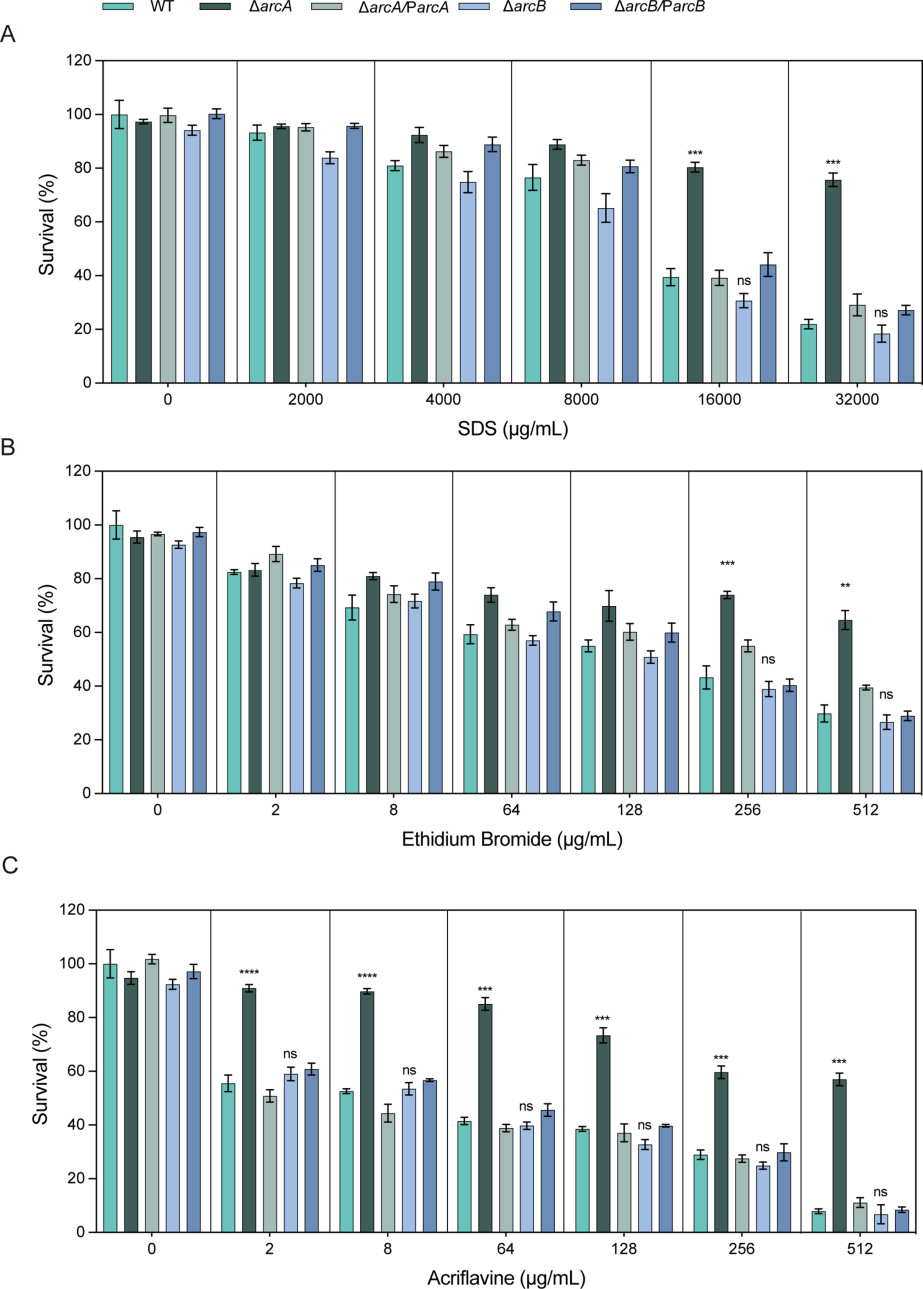
The susceptibility of the WT, Δ*arcA*, Δ*arcB*, Δ*arcA***/**P*arcA,* and Δ*arcB***/**P*arcB* strains to structurally related compounds. (**A**) Sensitivities of WT, Δ*arcA*, Δ*arcB*, Δ*arcA*/P*arcA,* and Δ*arcB*/P*arcB* strains to different concentrations (1,024 µg/mL, 2,048 µg/mL, 4,096 µg/mL, 8,192 µg/mL, 16,384 µg/mL) of SDS. The survival ability of the Δ*arcA* in SDS at 16,000 µg/mL was 1.88-fold and at 32,000 µg/mL was 3.45-fold higher compared to the WT strain. (**B**) Sensitivities of WT, Δ*arcA*, Δ*arcB*, Δ*arcA*/P*arcA,* and Δ*arcB*/P*arcB* strains to different concentrations (2 µg/mL, 8 µg/mL, 64 µg/mL, 128 µg/mL, 256 µg/mL, 512 µg/mL) of ethidium bromide. The survival ability of the Δ*arcA* in ethidium bromide at 256 µg/mL was 1.56-fold, and at 512 µg/mL was 1.54-fold compared to the WT strain. (**C**) Sensitivities of WT, Δ*arcA*, Δ*arcB*, Δ*arcA*/P*arcA,* and Δ*arcB*/P*arcB* strains to different concentrations (2 µg/mL, 8 µg/mL, 64 µg/mL, 128 µg/mL, 256 µg/mL, 512 µg/mL) of acriflavine. The survival ability of the Δ*arcA* in acriflavine at 2-, 8-, 64-, 128-, 256-, and 512 µg/mL was 1.50-, 1.60-, 1.84-, 1.90-, 1.92-, and 7.22-fold higher compared to the WT strain. The resistance percentages to different concentrations of SDS, ethidium bromide, and acriflavine were calculated by comparing the numbers of surviving cells to those in LB medium alone. Asterisks indicate statistically significant differences compared to the WT strain. Each experiment was performed in triplicate. *P < 0.05, **P < 0.01, ***P < 0.001, ****P < 0.0001, analyzed with one-way ANOVA.

When cells were exposed to varying concentrations of sodium dodecyl sulfate (SDS), it was observed that at 16,000 µg/mL, the CFU count of the Δ*arcA* was 1.88-fold higher than that of the wild type, and at 3,200 µg/mL, the CFU count of the Δ*arcA* was 3.45-fold higher than that of the wild type (Fig. 3A). Additionally, the susceptibility of the Δ*arcA* to ethidium bromide (EtBr) at 256 µg/mL and at 512 µg/mL (Fig. 3B) was decreased by 1.56-fold and 1.54-fold, respectively. Similarly, when cells were exposed to 2-, 8-, 64-, 128-, 256-, and 512 µg/mL acriflavine, the total CFU count of the Δ*arcA* was 1.50-, 1.60-, 1.84-, 1.90-, 1.92-, and 7.22-fold higher than that of the wild type (Fig. 3C). These results indicate that the deletion of *arcA* broadly affects the susceptibility of *K. pneumoniae* to various substances.

### Transcriptome analysis revealed that the upregulation of the gene of SMR *kpnEF* in the Δ*arcA* strain might be responsible for the reduced susceptibility

**Fig 4:**
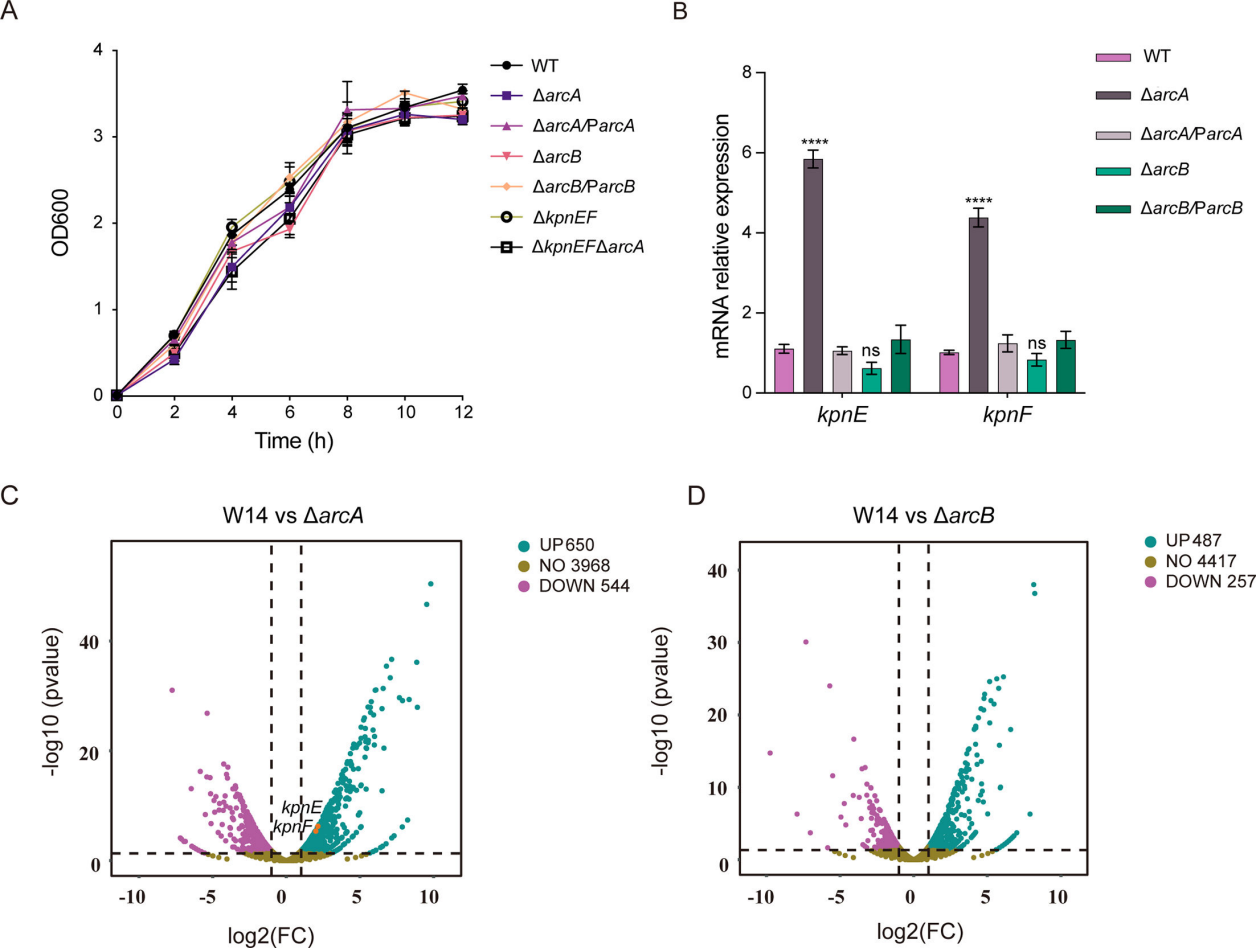
The regulation of ArcAB in *K. pneumoniae***.** The growth curves of WT, Δ*arcA*, Δ*arcA*/P*arcA*, Δ*arcB*, Δ*arcB*/P*arcB*, Δ*kpnEF,* and Δ*kpnEF*/Δ*arcA* strains (**A**). (**B**)The transcript levels of *kpnE* and *kpnF* in the WT, Δ*arcA*, Δ*arcB*, Δ*arcA*/P*arcA,* and Δ*arcB*/P*arcB* strains were detected by qRT-PCR. Volcano plot depicting gene transcripts to be differentially expressed between the WT and Δ*arcA* strains (**C**), WT and Δ*arcB* strains (**D**). Red and green dots represent significantly upregulated and downregulated proteins, respectively. ArcA negatively regulates the transcription of *kpnEF*. Error bars represent the standard errors of the means of three biological replicates. *P < 0.05, **P < 0.01, ***P < 0.001, ****P < 0.0001, analyzed with one-way ANOVA.

The growth curves of the WT, Δ*arcA*, Δ*arcB*, Δ*arcA*/P*arcA,* and Δ*arcB*/P*arcB* strains were determined to investigate whether the altered susceptibility of the Δ*arcA* to various compounds was growth-related. As shown in Fig. 4A, significant growth impairment in the Δ*arcA* and Δ*arcB* vs the WT (*P <* 0.05), no significant difference between the Δ*arcA* and Δ*arcB* strains (*P >* 0.05). To explore the regulatory mechanisms mediated by ArcA on bacterial responses to antibiotics, osmotic agents, disinfectants, and structurally related compounds, the global gene expression profiles of the WT, Δ*arcA*, and Δ*arcB* strains were analyzed using RNA-seq. Differentially expressed genes were identified based on an adjusted with *P* < 0.05 and |log2 (fold change) | >1. The results revealed that, among 5,406 genes, 1,194 were differentially expressed between the WT and Δ*arcA* strains and 744 between the WT and Δ*arcB* strains. Volcano plot analysis of the RNA-seq data showed that 544 genes were downregulated and 650 genes were upregulated in the Δ*arcA* (Fig. 4C), while 257 genes were downregulated and 487 genes were upregulated in the Δ*arcB* (Fig. 4D). Notably, the expression of the small multidrug efflux pump gene *kpnE and kpnF* was upregulated 4- and 3.71-fold in the Δ*arcA*, but no significant change was observed in the Δ*arcB*. The top 20 most upregulated and top 20 most downregulated genes in the Δ*arcA* compared to the wild-type and in the Δ*arcB* compared to the wild-type were showed in Tables S3 and S4. The qRT-PCR experiments further confirmed that the expression of *kpnE* and *kpnF* in the Δ*arcA* was 5.28- and 4.29-fold higher than that in the wild type, while the expression level in the Δ*arcA*/P*arcA* was restored to that of the wild type (Fig. 4B). In contrast, no significant changes in *kpnEF* expression were observed in the Δ*arcB* or the *arcB*/Δ*arcB* compared to the wild type (Fig. 4B). These findings suggested that ArcA regulates the expression of *kpnEF* independently of ArcB.

### The deletion of the *kpnEF* gene restored the antibiotic susceptibility of the Δ*arcA* strain

To investigate whether ArcA affected the antibiotic resistance of *K. pneumoniae* through the small multidrug efflux pump KpnEF, the mutant strains Δ*kpnEF* and Δ*kpnEF*Δ*arcA* were constructed by deleting *kpnEF* in the WT and Δ*arcA* background. Prior to MIC determination, growth curves were established for the WT, Δ*arcA*, Δ*kpnEF*, and Δ*kpnEF*Δ*arcA* strains. No significant differences were observed between the WT and Δ*kpnEF* strains, nor between the Δ*arcA* and Δ*kpnEF*Δ*arcA* strains (Fig. 4A). MIC assays revealed that the differential antibiotic susceptibility between the Δ*arcA* and WT strains was dependent on KpnEF, as deleting *kpnEF* in both backgrounds abolished this difference (Table 2). Additionally, Δ*kpnEF* alone showed significantly reduced resistance compared to WT, confirming the role of KpnEF as a key efflux pump contributing to intrinsic antibiotic resistance in this *K. pneumoniae*.

**TABLE 2:** The MICs of WT, Δ*arcA*, Δ*kpnEF,* and Δ*kpnEF***/**Δ*arcA* strains to antibiotics

	MIC (µg/mL)			
Antibiotics	WT	Δ*arcA*	Δ*kpnEF*	Δ*kpnEF* /Δ*arcA*
Cefepime	0.125	0.25	0.063	0.063
Ceftazidime	0.063	0.125	0.016	0.016
Kanamycin	0.625	2.5	0.031	0.031
Streptomycin	>62.5	>62.5	>62.5	>62.5
Ciprofloxacin	0.001	0.002	0.0005	0.0005
Colistin	1	4	0.5	0.5
Erythromycin	1.25	1.25	1.25	1.25
Polymyxin B	1	4	0.5	0.5
Rifampin	0.625	0.625	0.625	0.625
Tetracycline	3.125	6.25	1.563	1.563

### The deletion of *kpnEF* also restored the sensitivity of Δ*arcA* strain to osmotic stress, disinfectants, and structurally related compounds

**Fig 5:**
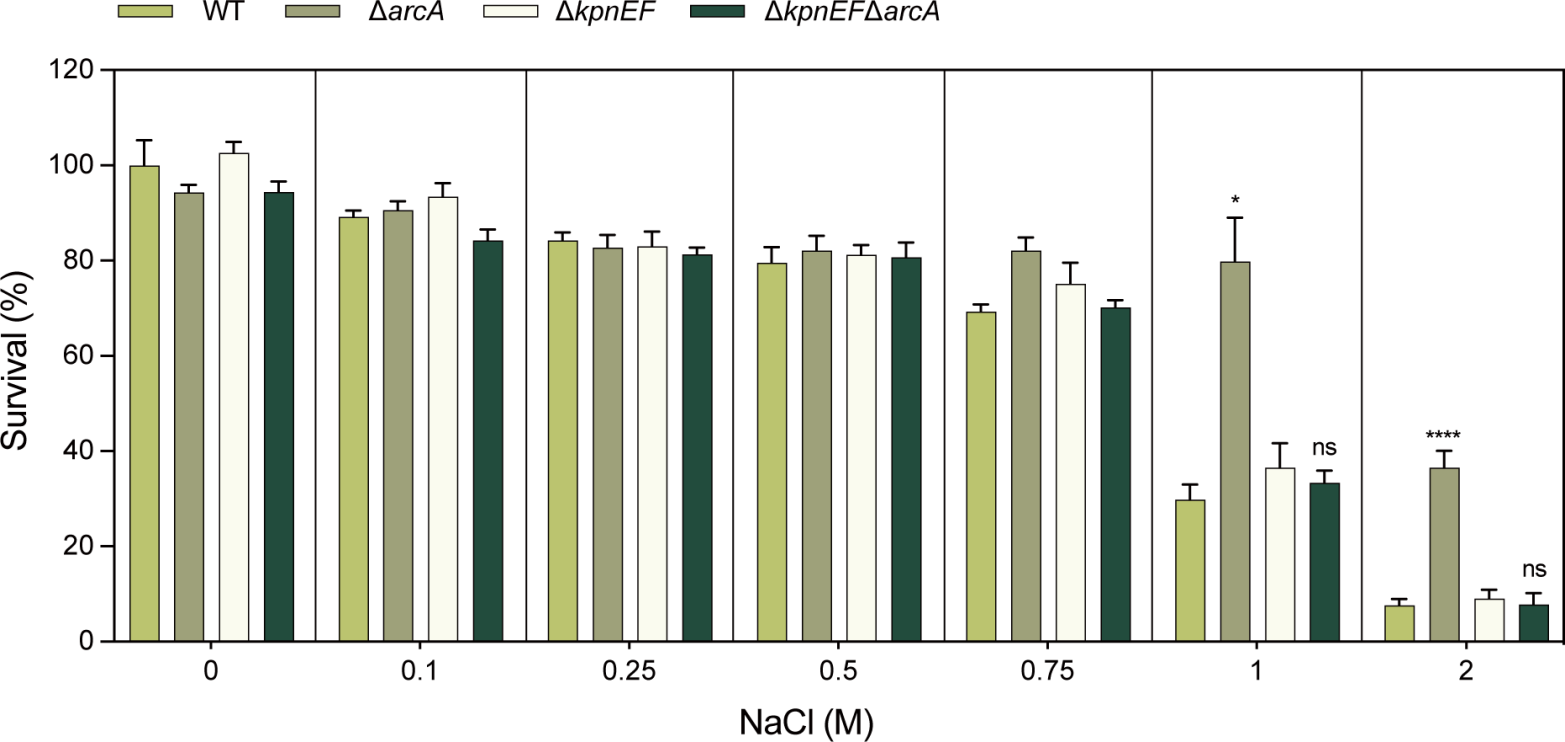
The osmotic stress agent susceptibility in the WT, Δ*arcA***,** Δ*kpnEF,* and Δ*kpnEF*Δ*arcA*strains. Sensitivities of the WT, Δ*arcA*, Δ*kpnEF,* and Δ*kpnEF*Δ*arcA* strains to different concentrations (0.075, 0.15, 0.25, 0.5, 0.75, 1.0, and 2.0 M) of NaCl. The MICs of the tested antibiotics showed no significant difference between Δ*kpnEF*Δ*arcA* and Δ*kpnEF* strains. Δ*kpnEF* exhibited significantly reduced antibiotic susceptibility compared to the WT strain. The tolerant percentages to different concentrations of NaCl were calculated by comparing the numbers of surviving cells to those in LB medium alone. *P < 0.05, **P < 0.01, ***P < 0.001, ****P < 0.0001, analyzed with one-way ANOVA.

**Fig 6:**
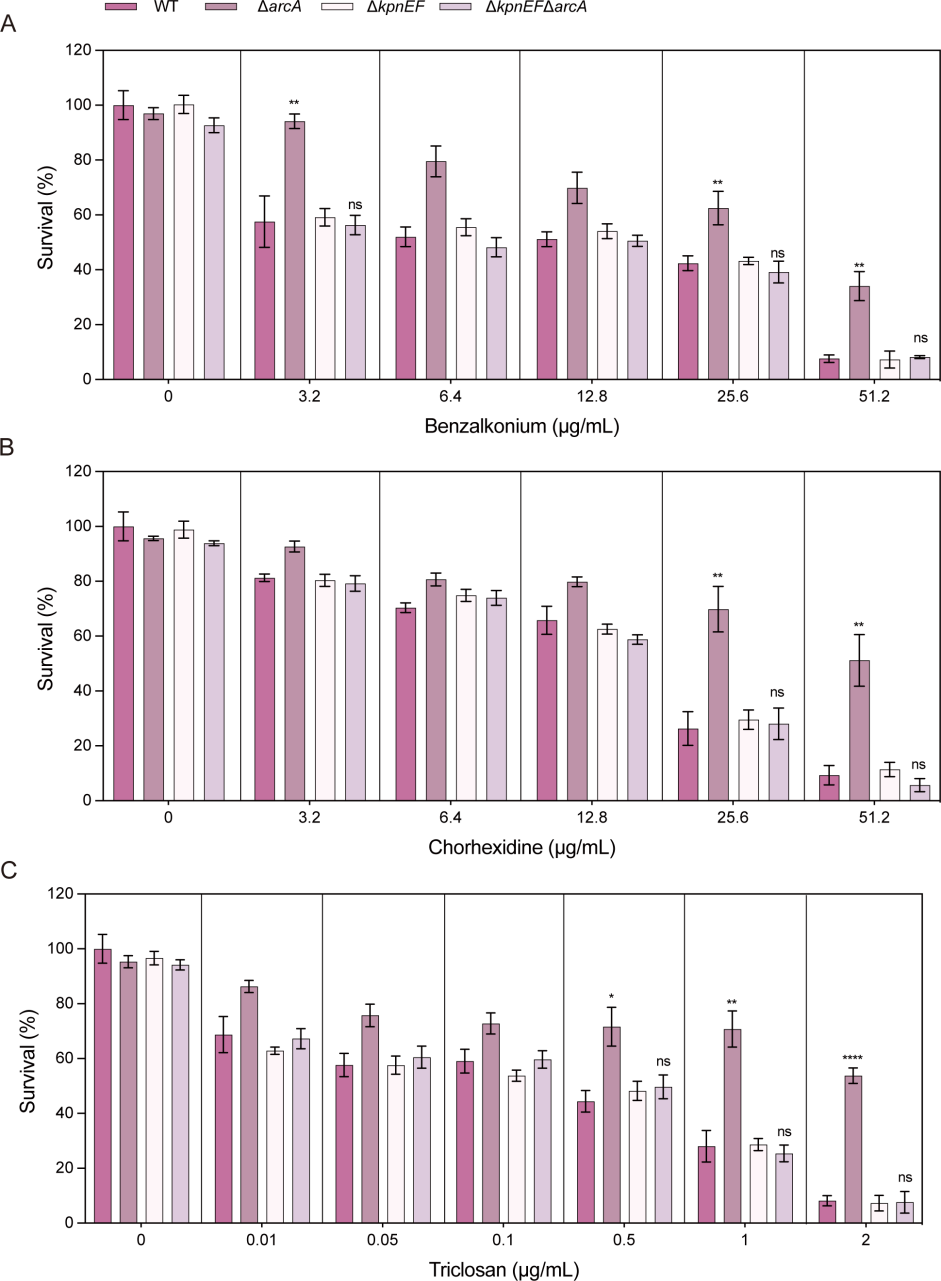
The hospital-based disinfectants susceptibility in the WT, Δ*arcA***,** Δ*kpnEF,* and Δ*kpnEF*Δ*arcA* strains**.** (**A**) Sensitivities of WT, Δ*arcA*, Δ*kpnEF,* and Δ*kpnEF*Δ*arcA* strains to different concentrations (3.2 µg/mL, 6.4 µg/mL, 12.8 µg/mL, 25.6 µg/mL, 51.2 µg/mL) of benzalkonium chloride. (**B**) Sensitivities of the WT, Δ*arcA*, Δ*kpnEF,* and Δ*kpnEF*Δ*arcA* strains to different concentrations (3.2 µg/mL, 6.4 µg/mL, 12.8 µg/mL, 25.6 µg/mL, 51.2 µg/mL) of chlorhexidine. (**C**) Sensitivities of the WT, Δ*arcA*, Δ*kpnEF,* and Δ*kpnEF*Δ*arcA* strains to different concentrations (0.01 µg/mL, 0.05 µg/mL, 0.1 µg/mL, 0.5 µg/mL, 1 µg/mL, 2 µg/mL) of triclosan. The sensitivity of Δ*kpnEF* mutant to these hospital-based disinfectants was not significantly different from that of Δ*kpnEF*Δ*arcA* mutant, while was significantly lower than that of WT strains. The tolerant percentages to different concentrations of benzalkonium chloride, chlorhexidine, and triclosan were calculated by comparing the numbers of surviving cells to those in LB medium alone. *P < 0.05, **P < 0.01, ***P < 0.001, ****P < 0.0001 analyzed with one-way ANOVA.

**Fig 7:**
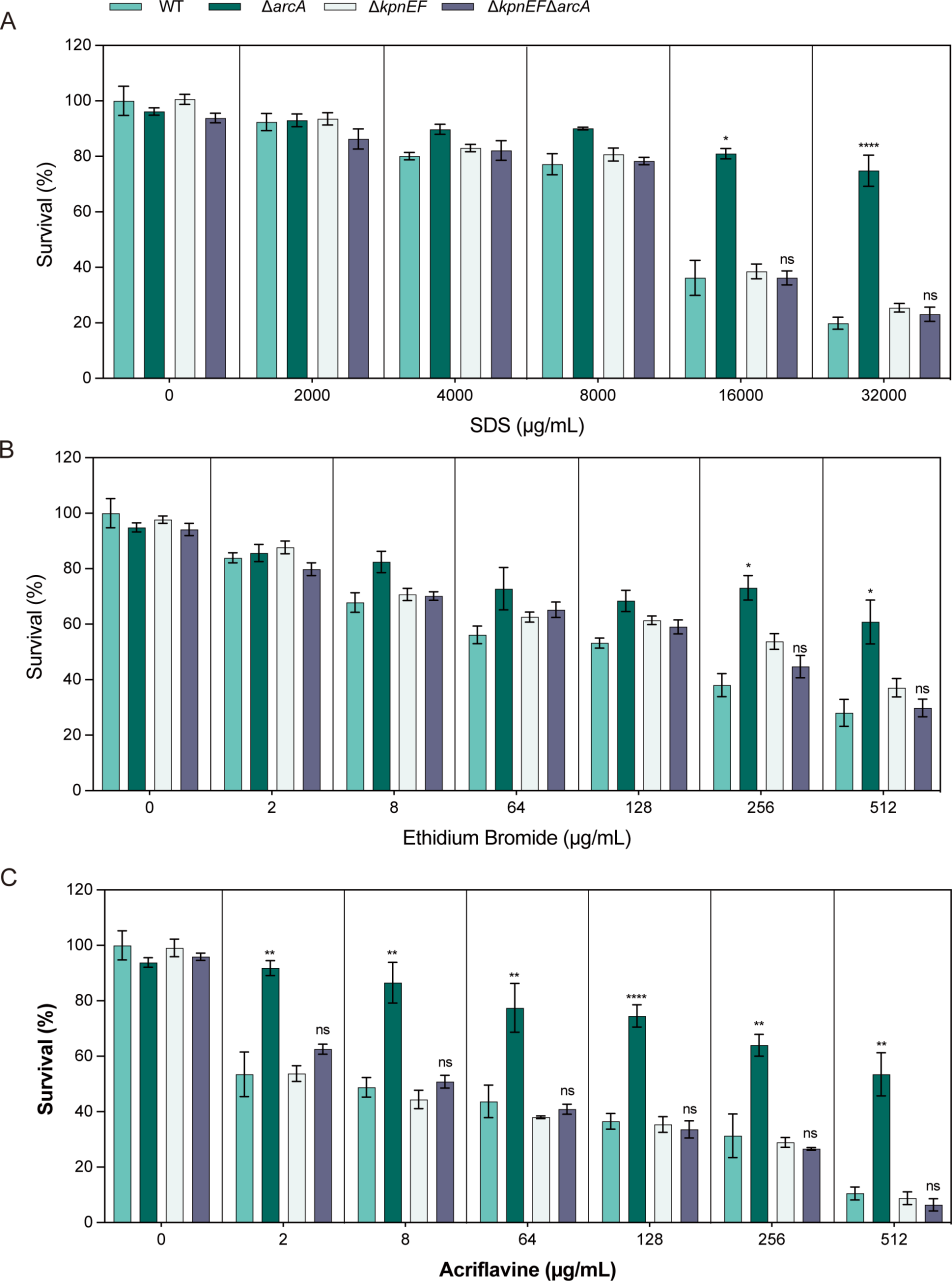
The structurally related compounds susceptibility in the WT, Δ*arcA***,** Δ*kpnEF,* and Δ*kpnEF*Δ*arcA* strains**.** (**A**) Sensitivities of WT, Δ*arcA*, Δ*kpnEF,* and Δ*kpnEF*Δ*arcA* strains to different concentrations (1,024 µg/mL, 2,048 µg/mL, 4,096 µg/mL, 8,192 µg/mL, 16,384 µg/mL) of SDS. (**B**) Sensitivities of the WT, Δ*arcA*, Δ*kpnEF,* and Δ*kpnEF*Δ*arcA* strains to different concentrations (2 µg/mL, 8 µg/mL, 64 µg/mL, 128 µg/mL, 256 µg/mL, 512 µg/mL) of ethidium bromide. (**C**) Sensitivities of the WT, Δ*arcA*, Δ*kpnEF,* and Δ*kpnEF*Δ*arcA* strains to different concentrations (2 µg/mL, 8 µg/mL, 64 µg/mL, 128 µg/mL, 256 µg/mL, 512 µg/mL) of acriflavine. The sensitivity of Δ*kpnEF* mutant to these structurally related compounds was not significantly different from that of Δ*kpnEF*Δ*arcA* mutant, while was significantly lower than that of WT strains. The tolerant percentages to different concentrations of SDS, ethidium bromide, and acriflavine were calculated by comparing the numbers of surviving cells to those in LB medium alone. *P < 0.05, **P < 0.01, *** *P <* 0.001, **** *P <* 0.0001, analyzed with one-way ANOVA.

To explore whether ArcA affected the susceptibility of *K. pneumoniae* to osmotic agents, disinfectants, and structurally related compounds through the small multidrug efflux pump KpnEF, the susceptibilities of the WT, Δ*arcA*, Δ*kpnEF,* and Δ*kpnEF*Δ*arcA* strains to varying concentrations of these compounds were determined. Under conditions of different concentrations of NaCl, benzalkonium chloride, chlorhexidine, triclosan, SDS, deoxycholate, EtBr, and acriflavine, the sensitivity of the Δ*kpnEF* to these compounds was not significantly different from that of the Δ*kpnEF*Δ*arcA*, while was significantly lower than that of wild-type strains. The results show that KpnEF influences the susceptibility of W14 to these compounds, and ArcA can influence the susceptibility of W14 to these compounds by mediating the expression of KpnEF (Fig. 5 to 7).

### ArcA ~*P* could specifically bind to the promoter region of *kpnEF* and repressed their expression, independently of ArcB

**Fig 8:**
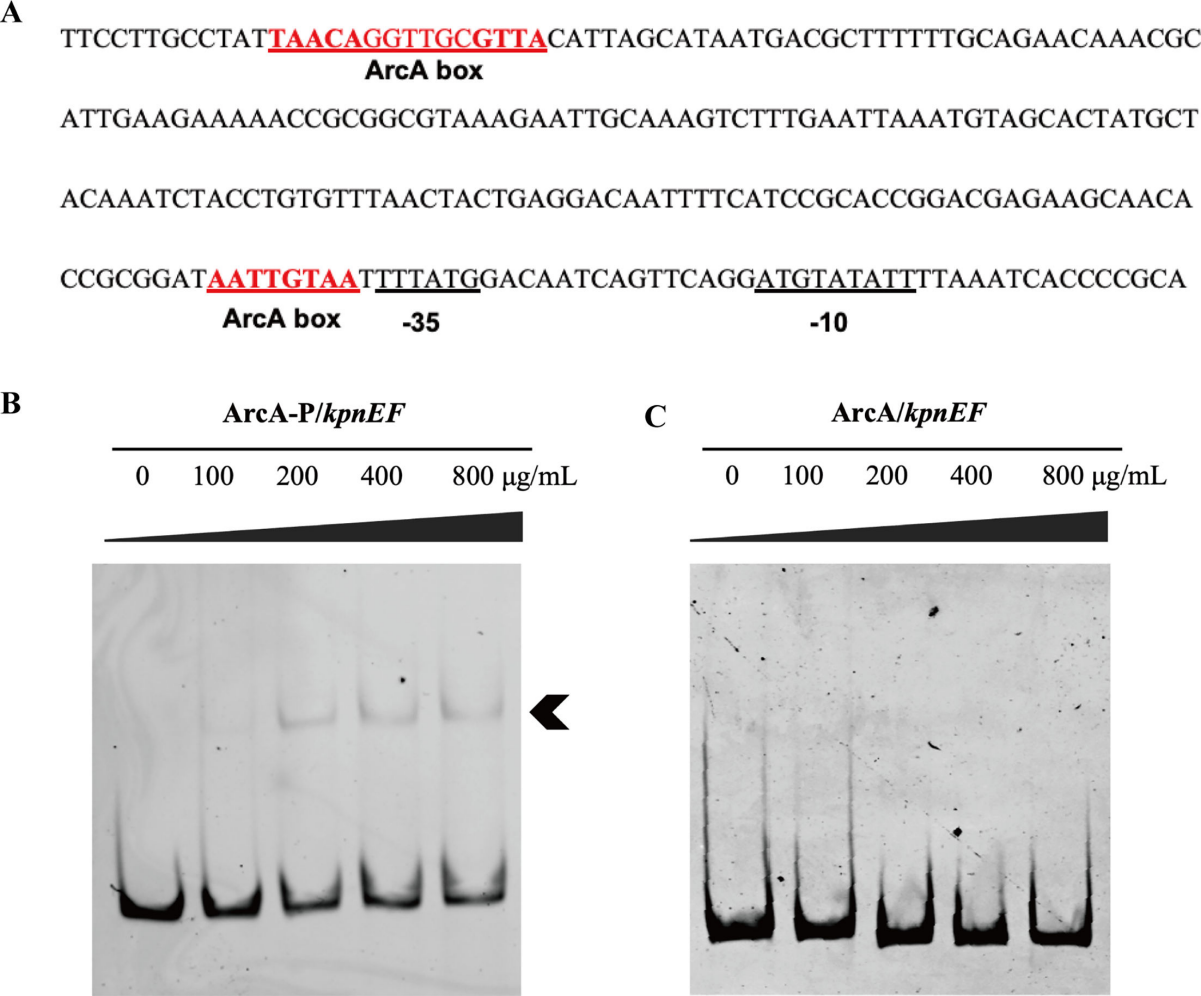
ArcA regulated the expression of the *kpnEF* operon by directly binding to their promoter regions. The promoter sequence of the *kpnEF* and the locations of the ArcA boxes were marked (**A**). The interactions between ArcA and the DNA fragments of their promoter regions were detected with EMSA. ArcA-P was incubated with the *kpnEF* promoter region (**B**), and ArcA was incubated with the *kpnEF* promoter region (**C**) at 37°C for 30 min. The mixtures were electrophoresed on 8% native polyacrylamide gels and the bands were imaged under UV light after EB staining.

Through upregulating SMR efflux pump KpnEF, the Δ*arcA* enhanced multidrug resistance and decreased susceptibility to osmotic agents, disinfectants, and structurally related compounds in *K. pneumoniae*. To determine whether ArcA regulated KpnEF by direct binding and whether phosphorylation of ArcA promotes its binding to the KpnEF promoter region, we conducted *in vitro* phosphorylation and the EMSA experiments. Based on previous reports, ArcA is known to bind to a conserved sequence (5′-TGTTA-3′) in *E. coli* (36). Subsequently, we used published consensus sequences and the Softberry bioinformatics platform to search for conserved ArcA binding sites in the *kpnEF* promoter region (37). Then the similar sequences (TAACA-GGTTGC-GTTA) and (AATTGTAA) located in the *kpnEF* promoter region were identified (Fig. 8A). The ArcA protein was expressed in *E. coli* BL21 using the pET-28a vector. EMSA was performed to detect the binding of ArcA and phosphorylated ArcA (ArcA-P) to a 574 bp DNA fragment of the *kpnEF* promoter region. As the amount of ArcA-P increased, the amount of free DNA gradually decreased (Fig. 8B). In contrast, the DNA mobility was unaffected when non-phosphorylated ArcA protein was used (Fig. 8C). These results indicate that ArcA requires phosphorylation to bind to the promoter region, and this process is independent of ArcB-mediated phosphorylation of ArcA, suggesting the existence of other proteins capable of phosphorylating ArcA.

### ArcA phosphorylation was mediated by the AckA-Pta pathway when ArcB was absent in *K. pneumoniae*

**Fig 9:**
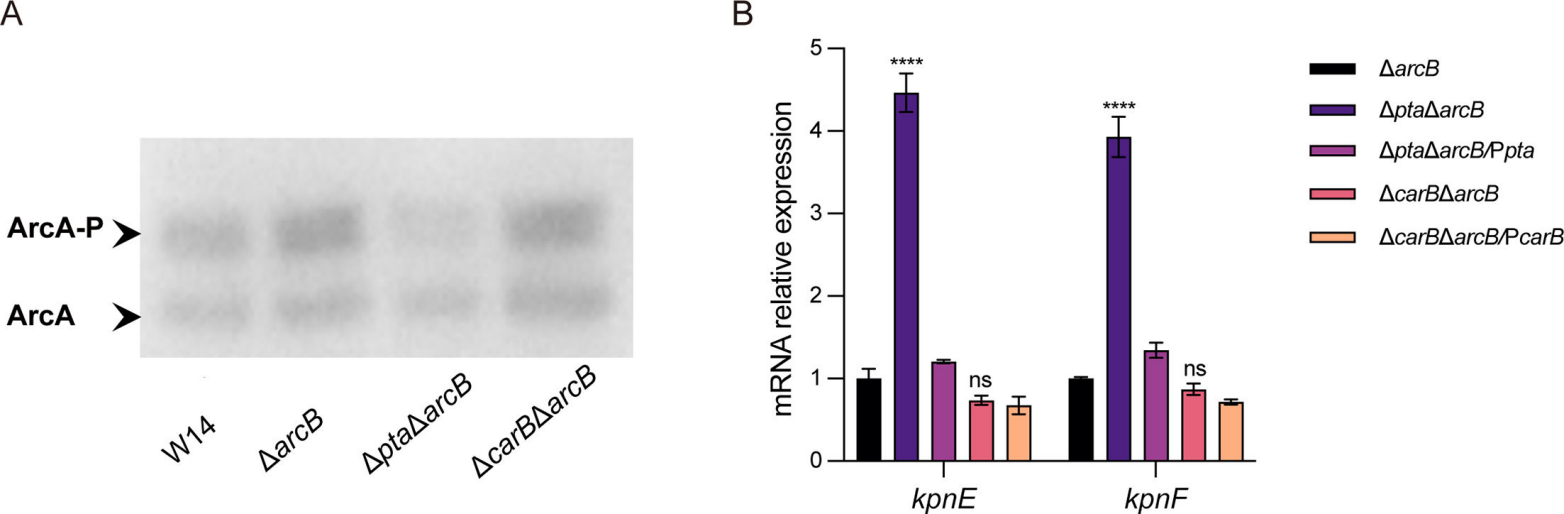
ArcA phosphorylation was mediated by the AckA-Pta pathway in the Δ*arcB* strain. (**A**) The phosphorylation levels of ArcA in the W14, Δ*arcB*, Δ*pta*Δ*arcB, and* Δ*carB*Δ*arcB* were performed by phos-assay. (**B**) The transcript levels of *kpnE* and *kpnF* in the Δ*arcB*, Δ*pta*Δ*arcB*, Δ*carB*Δ*arcB*, Δ*pta*Δ*arcB/pta,* and Δ*carB*Δ*arcB/carB* strains were detected by qRT-PCR. Error bars represent the standard errors of the means of three biological replicates. ****P < 0.0001 analyzed with one-way ANOVA.

Phosphorylation level assays demonstrated that the phosphorylation level of ArcA in the Δ*arcB* showed no significant difference compared to the wild-type, indicating the existence of alternative pathways mediating ArcA phosphorylation (Fig. 9A). The Co-IP experiment was performed to investigate whether other kinases could phosphorylate ArcA through direct binding. As shown in Table S5, the results were identified by adjusted −10 lgP ≥30 and unique ≥2. The kinase capable of directly binding and phosphorylating ArcA was not identified under these experimental conditions. Subsequently, the knockout strains of *pta* and *carB* genes were constructed in the Δ*arcB* background, respectively. Expression analysis of the *kpnEF* operon in these mutants demonstrated that deletion of *pta* significantly increased *kpnEF* expression (Fig. 9B). Phosphorylation assays further confirmed that the deletion of *pta* markedly decreased ArcA phosphorylation levels (Fig. 9A). These results demonstrate that in *K. pneumoniae*, ArcA could be phosphorylated via the AckA-Pta pathway under the absence of ArcB condition, enabling its regulatory function.

## DISCUSSION

The ArcAB system, also known as the Arc system, is a member of the bacterial two-component regulatory family, consisting of the sensor kinase ArcB and the response regulator ArcA. The ArcAB system exhibits broad regulatory capabilities, participating not only in central metabolism but also in a range of cellular processes, including bacterial conjugation, biofilm formation, stress response, acid tolerance, and even bioluminescence (38–42). ArcAB is predicted to orchestrate the sophisticated integration of these cellular processes with cognate metabolic pathways, thereby furnishing the energy and metabolic precursors essential for the execution of these functions. The regulatory role of ArcAB is particularly evident during infection, likely because pathogens must balance metabolic demands with survival in the host environment (36, 43, 44). In this study, we found that ArcA influenced sensitivity of *K. pneumoniae* to antibiotics, osmotic agents, disinfectants, and structural compounds by regulating the expression of the KpnEF multidrug efflux pump. This discovery enriched the regulatory network of the ArcAB system.

There is growing evidence that ArcA and ArcB do not always function physiologically as the “ArcAB system.” In *Serratia marcescens*, ArcB could regulate bacterial motility by activating the synthesis of flagella without regulator ArcA (45). In *Salmonella enterica*, ArcA was required in bacterial response to oxidative stress independent of its cognate sensor ArcB (46). And in this study, we found that the ArcA could regulate *kpnEF* independent of ArcB in *K. pneumoniae*. Thus, ArcA may act alone or in conjunction with other sensor protein to form another two-component system, while ArcB may also function with other regulator. Compared with other two-component systems, genes *arcA* and *arcB* are not found together in the same operon (47–49), which may explain the functional differences shown by ArcA and ArcB, since the expression and regulation of these two genes are relatively independent. Hence, the understanding of the function of ArcA and ArcB is far from enough.

Studies on the ArcA-DNA complex have revealed that ArcA typically bound to DNA fragments following dimerization or oligomerization (36, 50). The formation of higher-order ArcA oligomers is dependent on its phosphorylation status. Phosphorylated ArcA has been demonstrated to bind promoter regions at the −35 and −10 elements, as well as positions up to 500 bp upstream. The canonical ArcA-binding motif spans approximately 15 bp, with the most frequently observed sequence GTTAATTAAATGTTA being conserved across *E. coli*, *Salmonella*, and *Shewanella oneidensis*. Within this sequence are two direct repeats (GTTA) (51–54). In the current study, P-ArcA was found to bind to the promoter sequence of *kpnEF*, while non-phosphorylated ArcA showed no binding activity. This suggested that ArcA interacted with this fragment exclusively in its phosphorylated form. However, it remained unclear whether this binding occurred in a dimerized state. The phosphorylation level of ArcA primarily depended on the AckA-Pta pathway. Notably, the AckA-Pta pathway regulated acetyl phosphate (AcP) levels, which, in turn, influenced global protein acetylation, capsular polysaccharide (CPS) production, serum resistance, and type 3 fimbriae expression ultimately modulating virulence and playing a critical role in *K. pneumoniae* pathogenesis. In future studies, it will be essential to examine whether AcP levels are altered in the Δ*arcA* or Δ*arcB* and whether such changes affect the pathogenicity of *K. pneumoniae*.

## Data Availability

The raw sequence data of RNA-seq in this study have been deposited in the Genome Sequence Archive in National Genomics Data Center, Beijing Institute of Genomics (China National Center for Bioinformation), Chinese Academy of Sciences, under accession number CRA024048 (https://ngdc.cncb.ac.cn/gsub/submit/gsa/subCRA039074/finishedOverview), and are publicly accessible at https://bigd.big.ac.cn/gsa.

## SUPPLEMENTAL MATERIAL

Supplemental material spectrum.01499-25-s0001.docx Tables S1 to S5.

ASM does not own the copyrights to Supplemental Material that may be linked to, or accessed through, an article. The authors have granted ASM a non-exclusive, world-wide license to publish the Supplemental Material files. Please contact the corresponding author directly for reuse.

## References

[1] Wyres KL, Lam MMC, Holt KE. 2020. Population genomics of Klebsiella pneumoniae. Nat Rev Microbiol 18:344–359. doi:10.1038/s41579-019-0315-1 32055025

[2] Wyres KL, Holt KE. 2018. Klebsiella pneumoniae as a key trafficker of drug resistance genes from environmental to clinically important bacteria. Curr Opin Microbiol 45:131–139. doi:10.1016/j.mib.2018.04.004 29723841

[3] Yang X, Dong N, Chan EW-C, Zhang R, Chen S. 2021. Carbapenem resistance-encoding and virulence-encoding conjugative plasmids in Klebsiella pneumoniae. Trends Microbiol 29:65–83. doi:10.1016/j.tim.2020.04.012 32448764

[4] Aye SM, Galani I, Yu H, Wang J, Chen K, Wickremasinghe H, Karaiskos I, Bergen PJ, Zhao J, Velkov T, Giamarellou H, Lin YW, Tsuji BT, Li J. 2020. Polymyxin triple combinations against polymyxin-resistant, multidrug-resistant, KPC-producing Klebsiella pneumoniae. Antimicrob Agents Chemother 64:e00246-20. doi:10.1128/AAC.00246-20 32393492 PMC7526826

[5] Foster TJ. 2017. Antibiotic resistance in Staphylococcus aureus. Current status and future prospects. FEMS Microbiol Rev 41:430–449. doi:10.1093/femsre/fux007 28419231

[6] Piddock LJV. 2006. Multidrug-resistance efflux pumps - not just for resistance. Nat Rev Microbiol 4:629–636. doi:10.1038/nrmicro1464 16845433

[7] Piddock LJV. 2006. Clinically relevant chromosomally encoded multidrug resistance efflux pumps in bacteria. Clin Microbiol Rev 19:382–402. doi:10.1128/CMR.19.2.382-402.2006 16614254 PMC1471989

[8] Hassan KA, Maher C, Elbourne LD, Henderson PJ, Paulsen IT. 2021. Increasing the PACE of characterising novel transporters by functional genomics. Curr Opin Microbiol 64:1–8. doi:10.1016/j.mib.2021.08.005 34492595

[9] Paulsen IT. 2003. Multidrug efflux pumps and resistance: regulation and evolution. Curr Opin Microbiol 6:446–451. doi:10.1016/j.mib.2003.08.005 14572535

[10] Bay DC, Rommens KL, Turner RJ. 2008. Small multidrug resistance proteins: a multidrug transporter family that continues to grow. Biochim Biophys Acta 1778:1814–1838. doi:10.1016/j.bbamem.2007.08.015 17942072

[11] Chen YJ, Pornillos O, Lieu S, Ma C, Chen AP, Chang G. 2007. X-ray structure of EmrE supports dual topology model. Proc Natl Acad Sci USA 104:18999–19004. doi:10.1073/pnas.0709387104 18024586 PMC2141897

[12] Yelin R, Rotem D, Schuldiner S. 1999. EmrE, a small Escherichia coli multidrug transporter, protects Saccharomyces cerevisiae from toxins by sequestration in the vacuole. J Bacteriol 181:949–956. doi:10.1128/JB.181.3.949-956.1999 9922260 PMC93463

[13] Li XZ, Poole K, Nikaido H. 2003. Contributions of MexAB-OprM and an EmrE homolog to intrinsic resistance of Pseudomonas aeruginosa to aminoglycosides and dyes. Antimicrob Agents Chemother 47:27–33. doi:10.1128/AAC.47.1.27-33.2003 12499164 PMC149025

[14] Minato Y, Shahcheraghi F, Ogawa W, Kuroda T, Tsuchiya T. 2008. Functional gene cloning and characterization of the SsmE multidrug efflux pump from Serratia marcescens. Biol Pharm Bull 31:516–519. doi:10.1248/bpb.31.516 18310921

[15] Srinivasan VB, Rajamohan G, Gebreyes WA. 2009. Role of AbeS, a novel efflux pump of the SMR family of transporters, in resistance to antimicrobial agents in Acinetobacter baumannii. Antimicrob Agents Chemother 53:5312–5316. doi:10.1128/AAC.00748-09 19770280 PMC2786332

[16] Bay DC, Turner RJ. 2009. Diversity and evolution of the small multidrug resistance protein family. BMC Evol Biol 9:140. doi:10.1186/1471-2148-9-140 19549332 PMC2716321

[17] Masaoka Y, Ueno Y, Morita Y, Kuroda T, Mizushima T, Tsuchiya T. 2000. A two-component multidrug efflux pump, EbrAB, in Bacillus subtilis. J Bacteriol 182:2307–2310. doi:10.1128/JB.182.8.2307-2310.2000 10735876 PMC111282

[18] Srinivasan VB, Rajamohan G. 2013. KpnEF, a new member of the Klebsiella pneumoniae cell envelope stress response regulon, is an SMR-type efflux pump involved in broad-spectrum antimicrobial resistance. Antimicrob Agents Chemother 57:4449–4462. doi:10.1128/AAC.02284-12 23836167 PMC3754300

[19] Brown AN, Anderson MT, Bachman MA, Mobley HLT. 2022. The ArcAB two-component system: function in metabolism, redox control, and infection. Microbiol Mol Biol Rev 86:e0011021. doi:10.1128/mmbr.00110-21 35442087 PMC9199408

[20] Georgellis D, Lynch AS, Lin EC. 1997. In vitro phosphorylation study of the arc two-component signal transduction system of Escherichia coli. J Bacteriol 179:5429–5435. doi:10.1128/jb.179.17.5429-5435.1997 9286997 PMC179413

[21] Kwon O, Georgellis D, Lin EC. 2000. Phosphorelay as the sole physiological route of signal transmission by the Arc two-component system of Escherichia coli. J Bacteriol 182:3858–3862. doi:10.1128/JB.182.13.3858-3862.2000 10851007 PMC94563

[22] Teran-Melo JL, Peña-Sandoval GR, Silva-Jimenez H, Rodriguez C, Alvarez AF, Georgellis D. 2018. Routes of phosphoryl group transfer during signal transmission and signal decay in the dimeric sensor histidine kinase ArcB. J Biol Chem 293:13214–13223. doi:10.1074/jbc.RA118.003910 29945971 PMC6109937

[23] Lassak J, Henche AL, Binnenkade L, Thormann KM. 2010. ArcS, the cognate sensor kinase in an atypical Arc system of Shewanella oneidensis MR-1. Appl Environ Microbiol 76:3263–3274. doi:10.1128/AEM.00512-10 20348304 PMC2869118

[24] Liu X, Peña Sandoval GR, Wanner BL, Jung WS, Georgellis D, Kwon O. 2009. Evidence against the physiological role of acetyl phosphate in the phosphorylation of the ArcA response regulator in Escherichia coli. J Microbiol 47:657–662. doi:10.1007/s12275-009-0087-9 19851741

[25] Lynch AS, Lin EC. 1996. Transcriptional control mediated by the ArcA two-component response regulator protein of Escherichia coli: characterization of DNA binding at target promoters. J Bacteriol 178:6238–6249. doi:10.1128/jb.178.21.6238-6249.1996 8892825 PMC178496

[26] Drapal N, Sawers G. 1995. Purification of ArcA and analysis of its specific interaction with the pfl promoter-regulatory region. Mol Microbiol 16:597–607. doi:10.1111/j.1365-2958.1995.tb02422.x 7565118

[27] Federowicz S, Kim D, Ebrahim A, Lerman J, Nagarajan H, Cho B, Zengler K, Palsson B. 2014. Determining the control circuitry of redox metabolism at the genome-scale. PLoS Genet 10:e1004264. doi:10.1371/journal.pgen.1004264 24699140 PMC3974632

[28] Waegeman H, Beauprez J, Moens H, Maertens J, De Mey M, Foulquié-Moreno MR, Heijnen JJ, Charlier D, Soetaert W. 2011. Effect of iclR and arcA knockouts on biomass formation and metabolic fluxes in Escherichia coli K12 and its implications on understanding the metabolism of Escherichia coli BL21 (DE3). BMC Microbiol 11:70. doi:10.1186/1471-2180-11-70 21481254 PMC3094197

[29] Förster AH, Gescher J. 2014. Metabolic engineering of Escherichia coli for production of mixed-acid fermentation end products. Front Bioeng Biotechnol 2:16. doi:10.3389/fbioe.2014.00016 25152889 PMC4126452

[30] Yuan J, Chen C, Cui J, Lu J, Yan C, Wei X, Zhao X, Li N, Li S, Xue G, et al.. 2019. Fatty liver disease caused by high-alcohol-producing Klebsiella pneumoniae. Cell Metab 30:675–688. doi:10.1016/j.cmet.2019.08.018 31543403

[31] Link AJ, Phillips D, Church GM. 1997. Methods for generating precise deletions and insertions in the genome of wild-type Escherichia coli: application to open reading frame characterization. J Bacteriol 179:6228–6237. doi:10.1128/jb.179.20.6228-6237.1997 9335267 PMC179534

[32] Pan YJ, Fang HC, Yang HC, Lin TL, Hsieh PF, Tsai FC, Keynan Y, Wang JT. 2008. Capsular polysaccharide synthesis regions in Klebsiella pneumoniae serotype K57 and a new capsular serotype. J Clin Microbiol 46:2231–2240. doi:10.1128/JCM.01716-07 18508935 PMC2446917

[33] Hennequin C, Forestier C. 2009. oxyR, a LysR-type regulator involved in Klebsiella pneumoniae mucosal and abiotic colonization. Infect Immun 77:5449–5457. doi:10.1128/IAI.00837-09 19786563 PMC2786449

[34] Hellman LM, Fried MG. 2007. Electrophoretic mobility shift assay (EMSA) for detecting protein-nucleic acid interactions. Nat Protoc 2:1849–1861. doi:10.1038/nprot.2007.249 17703195 PMC2757439

[35] Coudeyras S, Nakusi L, Charbonnel N, Forestier C. 2008. A tripartite efflux pump involved in gastrointestinal colonization by Klebsiella pneumoniae confers a tolerance response to inorganic acid. Infect Immun 76:4633–4641. doi:10.1128/IAI.00356-08 18644883 PMC2546844

[36] Park DM, Akhtar MS, Ansari AZ, Landick R, Kiley PJ. 2013. The bacterial response regulator ArcA uses a diverse binding site architecture to regulate carbon oxidation globally. PLoS Genet 9:e1003839. doi:10.1371/journal.pgen.1003839 24146625 PMC3798270

[37] Shahmuradov IA, Gammerman AJ, Hancock JM, Bramley PM, Solovyev VV. 2003. PlantProm: a database of plant promoter sequences. Nucleic Acids Res 31:114–117. doi:10.1093/nar/gkg041 12519961 PMC165488

[38] Serna A, Espinosa E, Camacho EM, Casadesús J. 2010. Regulation of bacterial conjugation in microaerobiosis by host-encoded functions ArcAB and SdhABCD. Genetics 184:947–958. doi:10.1534/genetics.109.109918 20083612 PMC2865929

[39] Longo P, Ota-Tsuzuki C, Nunes A, Fernandes B, Mintz K, Fives-Taylor P, Mayer M. 2009. Aggregatibacter actinomycetemcomitans arcB influences hydrophobic properties, biofilm formation and adhesion to hydroxyapatite. Braz J Microbiol 40:550–562. doi:10.1590/S1517-838220090003000018 24031399 PMC3768537

[40] Cheng C, Chen J, Shan Y, Fang C, Liu Y, Xia Y, Song H, Fang W. 2013. Listeria monocytogenes ArcA contributes to acid tolerance. J Med Microbiol 62:813–821. doi:10.1099/jmm.0.055145-0 23518652

[41] Wang Z, Sun J, Xia T, Liu Y, Fu J, Lo YK, Chang C, Yan A, Liu X. 2018. Proteomic delineation of the ArcA regulon in Salmonella typhimurium during anaerobiosis. Mol Cell Proteomics 17:1937–1947. doi:10.1074/mcp.RA117.000563 30038032 PMC6166683

[42] Bose JL, Kim U, Bartkowski W, Gunsalus RP, Overley AM, Lyell NL, Visick KL, Stabb EV. 2007. Bioluminescence in Vibrio fischeri is controlled by the redox-responsive regulator ArcA. Mol Microbiol 65:538–553. doi:10.1111/j.1365-2958.2007.05809.x 17590235

[43] Iyer MS, Pal A, Srinivasan S, Somvanshi PR, Venkatesh KV. 2021. Global transcriptional regulators fine-tune the translational and metabolic efficiency for optimal growth of Escherichia coli. mSystems 6:e00001-21. doi:10.1128/mSystems.00001-21 33785570 PMC8546960

[44] Sun H, Song Y, Chen F, Zhou C, Liu P, Fan Y, Zheng Y, Wan X, Feng L. 2020. An ArcA-modulated small RNA in pathogenic Escherichia coli K1. Front Microbiol 11:574833. doi:10.3389/fmicb.2020.574833 33329434 PMC7719688

[45] Zhang X, Wu D, Guo T, Ran T, Wang W, Xu D. 2018. Differential roles for ArcA and ArcB homologues in swarming motility in Serratia marcescens FS14. Antonie Van Leeuwenhoek 111:609–617. doi:10.1007/s10482-017-0981-9 29139003

[46] Cabezas CE, Laulié AM, Briones AC, Pardo-Esté C, Lorca DE, Cofré AA, Morales EH, Mora AY, Krüger GI, Bueno SM, Hidalgo AA, Saavedra CP. 2021. Activation of regulator ArcA in the presence of hypochlorite in Salmonella enterica serovar Typhimurium. Biochimie 180:178–185. doi:10.1016/j.biochi.2020.11.009 33188860

[47] Groisman EA. 2016. Feedback control of two-component regulatory systems. Annu Rev Microbiol 70:103–124. doi:10.1146/annurev-micro-102215-095331 27607549 PMC8380452

[48] Capra EJ, Laub MT. 2012. Evolution of two-component signal transduction systems. Annu Rev Microbiol 66:325–347. doi:10.1146/annurev-micro-092611-150039 22746333 PMC4097194

[49] Yamamoto K, Hirao K, Oshima T, Aiba H, Utsumi R, Ishihama A. 2005. Functional characterization in vitro of all two-component signal transduction systems from Escherichia coli. J Biol Chem 280:1448–1456. doi:10.1074/jbc.M410104200 15522865

[50] Lu J, Peng Y, Wan S, Frost LS, Raivio T, Glover JNM. 2019. Cooperative function of TraJ and ArcA in regulating the F plasmid tra operon. J Bacteriol 201:e00448-18. doi:10.1128/JB.00448-18 PMC6287455 30322855

[51] Favorov AV, Gelfand MS, Gerasimova AV, Ravcheev DA, Mironov AA, Makeev VJ. 2005. A gibbs sampler for identification of symmetrically structured, spaced DNA motifs with improved estimation of the signal length. Bioinformatics 21:2240–2245. doi:10.1093/bioinformatics/bti336 15728117

[52] Wang X, Gao H, Shen Y, Weinstock GM, Zhou J, Palzkill T. 2008. A high-throughput percentage-of-binding strategy to measure binding energies in DNA-protein interactions: application to genome-scale site discovery. Nucleic Acids Res 36:4863–4871. doi:10.1093/nar/gkn477 18653527 PMC2528174

[53] Gao H, Wang X, Yang ZK, Palzkill T, Zhou J. 2008. Probing regulon of ArcA in Shewanella oneidensis MR-1 by integrated genomic analyses. BMC Genomics 9:42. doi:10.1186/1471-2164-9-42 18221523 PMC2262068

[54] Evans MR, Fink RC, Vazquez-Torres A, Porwollik S, Jones-Carson J, McClelland M, Hassan HM. 2011. Analysis of the ArcA regulon in anaerobically grown Salmonella enterica sv. Typhimurium. BMC Microbiol 11:58. doi:10.1186/1471-2180-11-58 21418628 PMC3075218

