# Supplementary material for: ArcA modulates multidrug resistance and compound susceptibility in Klebsiella pneumoniae through ArcB-independent regulation of the SMR efflux pump kpnEF
Source: Microbiol Spectr. 2025 Oct 13;13(11):e01499-25. doi: 10.1128/spectrum.01499-25 (PMC12584642; doi:10.1128/spectrum.01499-25)
Supplement: Supplemental material — Tables S1 to S5. [file spectrum.01499-25-s0001.docx]

**Supplementary Table 1** Bacterial strains and plasmids used in this study.

| **Strain/ Plasmid** | **Description** | **Source/Purpose** |
| --- | --- | --- |
| *K. pneumoniae* |  |  |
| W14 | Wild type strain of High-alcohol-producing  *K. pneumoniae* isolated from NAFLD&ABS patient | Jing Yuan *et al* [1]. |
| UAN | W14 deleted of *arcA* | This study |
| ∆*arcB* | W14 deleted of *arcB* | This study |
| ∆*kpnEF* | W14 deleted of *kpnEF* | This study |
| ∆*kpnEF*/∆*arcA* | W14 ∆*arcA* deleted of *kpnEF* | This study |
| ∆*arcA*/ *arcA* | Complemented *arcA* mutant with a pGEM-T Easy plasmid | This study |
| ∆*arcB*/ *arcB* | Complemented *arcB* mutant with a pGEM-T Easy plasmid | This study |
| Plasmid |  |  |
| pKO3-Km | Gene replacement plasmid derived from pKO3 with an insertion of Km resistance cassette into *AccI* site | Link *et al*[2]. |
| pKO3-*arcA* | pKO3 derivative, for *arcA* deletion | This study |
| pKO3-*arcB* | pKO3 derivative, for *arcB* deletion | This study |
| pKO3-*kpnEF* | pKO3 derivative, for *kpnEF* deletion | This study |
| pGEM-T-easy | Expression vector with an insertion of Km cassette | (Promega, Madison, WI, USA) |
| pGEM-T-easy-*arcA* | pGEM-T-easy derivative, for *arcA* mutant complementary | This study |
| pGEM-T-easy-*arcB* | pGEM-T-easy derivative, for *arcB* mutant complementary | This study |
| pET-28a | Expression vector with His tag in *E. coli*, Kmr | Addgene |
| pET-28a-*arcA* | His6-ArcA expression vector | This study |

**Supplementary Table 2** Primers used in this study.

| **Name** | **Sequence (5’-3’)** | **Purpose** |
| --- | --- | --- |
| KO-*arcA*-upF | TCGGTACCCGGGGATCGCATGGCGGAGGACAACAAGC | *arcA* deletion |
| KO-*arcA*-upR | AAGACGAGTTGGTAACACGCACATTCGTCGGATTCGTAAACAC |  |
| KO-*arcA*-dnF | GTGTTTACGAATCCGACGAATGTGCGTGTTACCAACTCGTCTT |  |
| KO-*arcA*-dnR | GGTCGACTCTAGAGGATCGCAGGTGGGCTTCGCTATCG |  |
| KO-*arcB*-upF | TCGGTACCCGGGGATCGCATCAAGATCGGTCCCAATGG | *arcB* deletion |
| KO-*arcB*-upR | GCCTGGTCTGATAATGTCGCT |  |
| KO-*arcB*-dnF | AGCCAACAGCATCGAAAAGC |  |
| KO-*arcB*-dnR | GGTCGACTCTAGAGGATCGCGGATGAAGACGGATATGAACTACG |  |
| KO-*kpnEF*-upF | TCGGTACCCGGGGATCGCACACCACCGTCAGCAGCAGCAG | *kpnEF* deletion |
| KO-*kpnEF*-upR | CACCATGCCGGCTACCAGCAGGATTTGCCCATTTCATAGACAAGGTGC |  |
| KO-*kpnEF*-dnF | GCACCTTGTCTATGAAATGGGCAAATCCTGCTGGTAGCCGGCATGGTG |  |
| KO-*kpnEF*-dnR | GGTCGACTCTAGAGGATCGCTCACGATGACCGTAAAGCGGTGAAC |  |
| KO-*pta*-upF | TCGGTACCCGGGGATCGCTACAGCCTGTACAAAGAGCAC |  |
| KO-*pta*-upR | TAGACGATATCGTCTACCAGGAACGCCTTTGCGTTCCAT |  |
| KO-*pta*-dnF | ATGGAACGCAAAGGCGTTCCTGGTAGACGATATCGTCTA |  |
| KO-*pta*-dnR | GGTCGACTCTAGAGGATCGCTGCTGCTTCGTTTTCCAATG |  |
| KO-*carB*-upF | TCGGTACCCGGGGATCGCAACCTATAGCTGGACGCAAGG |  |
| KO- *carB*-upR | CTGAATGTGCGGACGACCTTCATGGTTAGAGTTCACCAGGATAA |  |
| KO- *carB*-dnF | TTATCCTGGTGAACTCTAACCATGAAGGTCGTCCGCACATTCAG |  |
| KO- *carB*-dnR | GGTCGACTCTAGAGGATCGCTTGTCCATCCACAGCTTGAGCAG |  |
| C- *arcA*-TeasyF | AATTGGGCCCGACGTCGCATGCTCAGGGACTTTGGTACTTCC | Expression of *arcA* in Kpn |
| C- *arcA*-TeasyR | TTGGGAGCTCTCCCATATGGTCGACAAGCTGCGTCTCTGGAAAGAG |  |
| C- *arcB*-TeasyF | AATTGGGCCCGACGTCGCATGCTTGCACAACTTACAGCGCATTGC | Expression of *arcB* in Kpn |
| C- *arcB*-TeasyR | TTGGGAGCTCTCCCATATGGTCGACACATCACTCAAACAACACTGG |  |
| C- *kpnEF*-TeasyF | GGGTTTTCCCAGTCACGACACGTCTCGCGTAAACGGTAATGG | Expression of *kpnEF* in Kpn |
| C- *kpnEF*-TeasyR | TGCAATGTAACATCAGAGATTTTGTAGGCGTGAAGGAAGCTTCCGTTTG |  |
| C-*pta*-TeasyF | AATTGGGCCCGACGTCGCATGCTTTGAAAGATGACGGATAGCTG |  |
| C- *pta*-TeasyR | TTGGGAGCTCTCCCATATGGTCGACAATCGTCGCTCGCCTCCTGACGAC |  |
| C- *carB*-TeasyF | AATTGGGCCCGACGTCGCATGCTTGTAAACTGCGCTTTCTCAC |  |
| C- *carB*-TeasyR | TTGGGAGCTCTCCCATATGGTCGACAAAGCAGAACTCTCTGAAGATG |  |
| *rpoB*-RT-F | TGCAATACGTCAGCTACCGTCTG | qRT-PCR |
| *rpoB-*RT-R | TTGATAACAAAGGTACCGTTGTC |  |
| *kpnE*- RT-F | ACCTTGTCTATGAAATGGGCAAGC |  |
| *kpnE*- RT-R | AGCACGCTGAACAGGGTAATCAG |  |
| *kpnF*- RT-F | AAGATCTACGGCATTCTGTCC |  |
| *kpnF*- RT-R | AACACCATGCCGGCTACCAGCAGG |  |
| pET28a-*arcA*-F | ATGGGTCGCGGATCCGAATTCCAGACCCCGCACATTCTTATCG | Expression of  *arcA* in *E. coli* |
| pET28a-*arcA*-R | TTGTCGACGGAGCTCGAATTCAAGCTGCGTCTCTGGAAAGAG |  |
| E-*kpnEF*-F | AGGATGATCACCAGCATGCCCAAC | EMSA |
| E-*kpnEF*-R | TAGACAAGGTGCCGGTAATTTCAGC | EMSA |

**Supplemental Table 3** The top 20 most upregulated and top 20 most downregulated genes in the Δ*arcA* compared to the wild-type.

| **Gene_name** | **Log2 FoldChange** | **Gene_description** |
| --- | --- | --- |
| A7321_21745 | 9.812 | urocanate hydratase |
| A7321_21750 | 9.535 | histidine ammonia-lyase |
| A7321_22905 | 8.900 | gamma-glutamylputrescine oxidoreductase |
| A7321_11925 | 8.859 | 4-aminobutyrate transaminase |
| proY | 8.342 | proline-specific permease ProY |
| A7321_17185 | 8.237 | 2-hydroxyhepta-2%2C4-diene-1%2C7-dioate isomerase |
| A7321_22910 | 7.890 | aldehyde dehydrogenase PuuC |
| A7321_15305 | 7.876 | hypothetical protein |
| A7321_02845 | 7.683 | iron ABC transporter substrate-binding protein |
| A7321_19560 | 7.319 | 3-oxosteroid 1-dehydrogenase |
| A7321_19565 | 7.160 | shikimate dehydrogenase |
| A7321_12885 | 7.154 | aquaporin |
| A7321_15315 | 7.058 | acetate--CoA ligase |
| A7321_07365 | 6.802 | alpha-ketoglutarate transporter |
| A7321_23995 | 6.776 | succinylarginine dihydrolase |
| A7321_02200 | 6.631 | LysR family transcriptional regulator |
| A7321_22925 | 6.579 | gamma-glutamylputrescine synthetase |
| A7321_17155 | 6.537 | 2%2C4-dihydroxyhept-2-ene-1%2C7-dioic acid aldolase |
| ssuC | 6.537 | alkanesulfonate transporter permease subunit |
| A7321_18900 | 6.502 | carbon starvation induced protein |
| A7321_24935 | -5.556 | 2'-hydroxyisoflavone reductase |
| A7321_02070 | -5.556 | spermidine/putrescine ABC transporter permease |
| A7321_02650 | -5.556 | creatinine amidohydrolase |
| A7321_01190 | -5.556 | diguanylate cyclase |
| A7321_11540 | -5.774 | hypothetical protein |
| A7321_26585 | -5.774 | hypothetical protein |
| A7321_00180 | -5.845 | fumarate hydratase |
| A7321_18855 | -5.964 | oxidoreductase |
| A7321_24425 | -5.964 | hypothetical protein |
| A7321_08205 | -6.131 | hemin transporter |
| A7321_03940 | -6.131 | formate acetyltransferase |
| psiB | -6.131 | plasmid SOS inhibition protein B |
| A7321_02505 | -6.281 | hypothetical protein |
| A7321_08805 | -6.417 | ethanolamine utilization protein EutM |
| A7321_07675 | -6.458 | fimbrial assembly protein |
| A7321_26175 | -6.860 | type IV conjugative transfer system protein TraV |
| pduE | -6.952 | propanediol dehydratase |
| A7321_08840 | -7.121 | ethanolamine utilization protein EutM |
| A7321_26190 | -7.198 | type IV conjugative transfer system protein TraE |
| A7321_07680 | -7.753 | fimbrial protein |

**Supplemental Table 4** The top 20 most upregulated and top 20 most downregulated genes in the Δ*arcB* compared to the wild-type.

| **Gene_name** | **Log2 FoldChange** | **Gene_description** |
| --- | --- | --- |
| A7321_21750 | 8.225 | histidine ammonia-lyase |
| A7321_21745 | 8.154 | urocanate hydratase |
| A7321_25255 | 7.903 | succinylglutamate desuccinylase |
| A7321_19225 | 7.002 | hypothetical protein |
| A7321_05030 | 6.811 | tRNA-Asn |
| rprA | 6.705 | antisense sRNA RprA |
| A7321_23995 | 6.590 | succinylarginine dihydrolase |
| A7321_18340 | 6.590 | type II secretion system protein GspG |
| proY | 6.588 | proline-specific permease ProY |
| A7321_17155 | 6.466 | 2%2C4-dihydroxyhept-2-ene-1%2C7-dioicacid aldolase |
| A7321_16035 | 6.330 | sugar:proton symporter |
| A7321_00665 | 6.180 | sodium:solute symporter |
| A7321_23170 | 6.180 | hypothetical protein |
| A7321_00445 | 6.180 | hypothetical protein |
| A7321_21660 | 6.099 | hypothetical protein |
| A7321_19565 | 6.013 | shikimate dehydrogenase |
| A7321_03785 | 6.013 | sulfatase modifying factor 1 |
| A7321_21440 | 6.013 | hypothetical protein |
| A7321_22905 | 5.902 | gamma-glutamylputrescine oxidoreductase |
| A7321_25250 | 5.881 | succinylarginine dihydrolase |
| A7321_02505 | -3.188 | hypothetical protein |
| A7321_20175 | -3.298 | lysine decarboxylase LdcC |
| A7321_13055 | -3.324 | aminoglycoside/multidrug transporter permease |
| A7321_08805 | -3.324 | ethanolamine utilization protein EutM |
| A7321_07665 | -3.448 | fimbrial protein |
| A7321_19065 | -3.508 | fimbrial protein |
| A7321_08720 | -3.563 | cobalt ECF transporter T component CbiQ |
| A7321_19070 | -3.699 | hypothetical protein |
| A7321_09750 | -4.066 | helix-turn-helix transcriptional regulator |
| A7321_07670 | -4.107 | export and assembly usher protein of type 1 fimbriae |
| A7321_08845 | -4.610 | microcompartment protein |
| pduC | -4.716 | propanediol dehydratase |
| pduD | -4.941 | propanediol dehydratase |
| A7321_19075 | -5.503 | hypothetical protein |
| A7321_00675 | -5.842 | alcohol dehydrogenase |
| A7321_26585 | -5.842 | hypothetical protein |
| pduE | -7.021 | propanediol dehydratase |
| A7321_07680 | -7.322 | fimbrial protein |
| A7321_08810 | -7.923 | microcompartment protein PduB |
| A7321_07675 | -9.753 | fimbrial assembly protein |

**Supplementary Table 5** Proteins potentially interacting with ArcA identified by Co-IP/MS in the Δ*arcB* strain.

| **Protein** | **-10lgP** | **Coverage (%)** | **Peptides** | **Unique** | **Function** |
| --- | --- | --- | --- | --- | --- |
| ArcA | 225.15 | 47 | 13 | 13 | Aerobic respiration control protein ArcA |
| GuaA | 72.53 | 13 | 4 | 4 | GMP synthase |
| FadI | 54.01 | 8 | 2 | 2 | 3-ketoacyl-CoA thiolase |
| GlnS | 53 | 4 | 2 | 2 | Glutamine--tRNA ligase |
| RibF | 41.87 | 6 | 2 | 2 | Riboflavin biosynthesis protein |
| GlmU | 39.59 | 5 | 3 | 3 | Bifunctional protein GlmU |
| MdtO | 36.45 | 4 | 3 | 3 | Multidrug resistance protein MdtO |
| NudJ | 36.45 | 14 | 2 | 2 | Phosphatase NudJ |
| KPK_3325 | 35.15 | 3 | 2 | 2 | 3-octaprenyl-4-hydroxybenzoate carboxy-lyase family protein |
| KPK_2535 | 35.03 | 10 | 2 | 2 | ATPase AFG1 family |
| PbpC | 33.84 | 6 | 3 | 3 | peptidoglycan glycosyltransferase |
| MalI | 32.54 | 8 | 3 | 2 | Maltose regulon regulatory protein |
| PflA | 32.38 | 14 | 3 | 3 | Pyruvate formate-lyase-activating enzyme |
| KPK_0937 | 32.22 | 3 | 2 | 2 | Type VI secretion protein VC_A0111 family |
| MlaC | 31.12 | 13 | 2 | 2 | Intermembrane phospholipid transport system binding protein |

1. Yuan, J.; Chen, C.; Cui, J.; Lu, J.; Yan, C.; Wei, X.; Zhao, X.; Li, N.; Li, S.; Xue, G.; et al. Fatty Liver Disease Caused by High-Alcohol-Producing Klebsiella pneumoniae. *Cell Metab* **2019**, *30*, 675-688.e677, doi:10.1016/j.cmet.2019.08.018.

2. Link, A.J.; Phillips, D.; Church, G.M. Methods for generating precise deletions and insertions in the genome of wild-type Escherichia coli: application to open reading frame characterization. *J Bacteriol* **1997**, *179*, 6228-6237, doi:10.1128/jb.179.20.6228-6237.1997.
